# Pyrrolidine in Drug Discovery: A Versatile Scaffold for Novel Biologically Active Compounds

**DOI:** 10.1007/s41061-021-00347-5

**Published:** 2021-08-10

**Authors:** Giovanna Li Petri, Maria Valeria Raimondi, Virginia Spanò, Ralph Holl, Paola Barraja, Alessandra Montalbano

**Affiliations:** 1 Department of Biological, Chemical and Pharmaceutical Sciences and Technologies (STEBICEF), University of Palermo, Via Archirafi 32, 90123 Palermo, Italy; 2 Department of Chemistry, Institute of Organic Chemistry, University of Hamburg, Martin-Luther-King-Platz 6, 20146 Hamburg, Germany

**Keywords:** Pyrrolidine, Anticancer and antibacterial agents, Central nervous system diseases, Antidiabetics, Anti-inflammatory and analgesic agents

## Abstract

The five-membered pyrrolidine ring is one of the nitrogen heterocycles used widely by medicinal chemists to obtain compounds for the treatment of human diseases. The great interest in this saturated scaffold is enhanced by (1) the possibility to efficiently explore the pharmacophore space due to sp^3^-hybridization, (2) the contribution to the stereochemistry of the molecule, (3) and the increased three-dimensional (3D) coverage due to the non-planarity of the ring—a phenomenon called “pseudorotation”. In this review, we report bioactive molecules with target selectivity characterized by the pyrrolidine ring and its derivatives, including pyrrolizines, pyrrolidine-2-one, pyrrolidine-2,5-diones and prolinol described in the literature from 2015 to date. After a comparison of the physicochemical parameters of pyrrolidine with the parent aromatic pyrrole and cyclopentane, we investigate the influence of steric factors on biological activity, also describing the structure–activity relationship (SAR) of the studied compounds. To aid the reader’s approach to reading the manuscript, we have planned the review on the basis of the synthetic strategies used: (1) ring construction from different cyclic or acyclic precursors, reporting the synthesis and the reaction conditions, or (2) functionalization of preformed pyrrolidine rings, e.g., proline derivatives. Since one of the most significant features of the pyrrolidine ring is the stereogenicity of carbons, we highlight how the different stereoisomers and the spatial orientation of substituents can lead to a different biological profile of drug candidates, due to the different binding mode to enantioselective proteins. We believe that this work can guide medicinal chemists to the best approach in the design of new pyrrolidine compounds with different biological profiles.

## Introduction

The development of clinically active drugs relies increasingly on the use of heterocyclic scaffolds, many of which contain nitrogen, as evidenced by the considerable number of bioactive compounds now available [1–5]. The introduction of heteroatomic fragments in these molecules is not a random choice, considering that they are useful tools for modifying physicochemical parameters and obtaining the best ADME/Tox results for drug candidates [6, 7]. Although two-dimensional (2D) flat heteroaromatic ring scaffolds are used widely by medicinal chemists, due mainly to their easy structural modification [8, 9], heteroatomic saturated ring systems allow a greater chance of generating structural diversity [10]. This molecular complexity was defined by Lovering et al. [11] through two descriptors, the sp^3^-hybridization and number of chiral centers, both of which are essential for establishing the clinical success of new bioactive molecules. Indeed, the chemical complexity and the globular three-dimensional (3D) shape offer more opportunities to improve druggability by modifying parameters such as solubility, lipophilicity, and other ADME properties [12]. This association was also highlighted by Ritchie et al. [13] who conducted an interesting study on a set of 19,196 molecules containing three rings with different degrees of aromaticity. Molecules with growing hetero-aliphatic character showed an increase in aqueous solubility compared with those comprising three aromatic rings. Thus, each portion of the molecules can potentially influence the balance of the physicochemical parameters, which needs to be considered when designing compounds with an optimized pharmacokinetic (PK) profile. Table 1 summarizes a comparison of some of the molecular descriptors and parameters of the pyrrolidine nucleus with that of carbocyclic cyclopentane and aromatic pyrrole. As shown in Table 1, the presence of the nitrogen atom contributes to the polarity of the molecules, producing a dipole moment (*D*) and a marked PSA value, which cyclopentane lacks. Although both, pyrrole and pyrrolidine, show similar polar surface area (PSA) values, only the pK_BHX_ values (pyrrole = 0.15, pyrrolidine = 2.59) take into account the strength of the H-bonds. The presence of nitrogen also affects the lipophilicity of the heterocyclic rings, as evidenced by the lower LogP values of pyrrolidine and pyrrole, and the solvent accessible surface area (SASA) FISA and FOSA values. Also interesting is the CI_logS value of pyrrole, which is different from the LogS, highlighting how the planarity of the aromatic ring can influence aqueous solubility.

**Table 1:** Comparison of molecular descriptors and parameters of the pyrrolidine nucleus with those of carbocyclic cyclopentane and aromatic pyrrole

Molecule	*D*	SASA (Å^2^)	FOSA	FISA	PISA	DonorHB	AcceptHB	LogPo/w	LogS	CI_LogS	pK_BHX_	PSA
Cyclopentane	0.073	269.230	269.230	0	0	0	0	3.000	−2.642	−2.709	0	0
Pyrrolidine	1.411	258.835	225.518	33.317	0	1.000	1.500	0.459	0.854	0.809	2.59	16.464
Pyrrole	2.930	236.257	0	31.512	204.745	1.000	0.500	0.750	−0.175	−0.542	0.15	13.964

*D* Computed dipole moment of the molecule, *SASA* total solvent accessible surface area using a probe with a 1.4 Å radius,* FOSA* hydrophobic component of the SASA (saturated carbon and attached hydrogen);* FISA*, hydrophilic component of the SASA (SASA on N, O, and H on heteroatoms),* PISA* carbon and attached hydrogen component of the SASA,* DonorHB* estimated number of hydrogen bonds donated by the solute to water molecules in aqueous solution (values are averages taken over a number of configurations, so they can be non-integer),* AcceptHB* estimated number of hydrogen bonds accepted by the solute from water molecules in aqueous solution (values are averages taken over a number of configurations, so they can be non-integer),* LogPo/w* predicted octanol/water partition coefficient,* LogS* predicted aqueous solubility (S in mol dm^−3^ is the concentration of the solute in a saturated solution that is in equilibrium with the crystalline solid),* CI_LogS* conformation-independent predicted aqueous solubility,* pK*_*BHX*_ hydrogen-bond basicity scale, *PSA* Van der Waals surface area of polar nitrogen and oxygen atoms. Molecular descriptors and parameters were calculated using Qikprop software (version 6.2, Schrödinger, LLC, New York, NY, 2019)

**Fig. 1:**
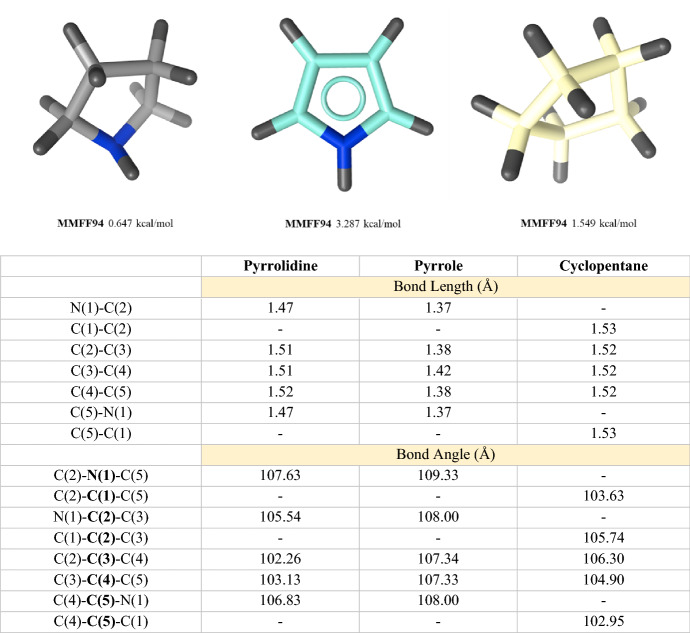
Comparison of the three-dimensional (3D) shape of the non-aromatic pyrrolidine and cyclopentane rings with the aromatic pyrrole ring. Bond angles, bond lengths, and MMFF94 values were calculated using LigandScout software version 4.4 Expert

Over the past two decades, much effort have been made by industry to provide the chemistry community with new sp^3^-enriched 3D building blocks as commercial sources from which to produce molecules relevant from a medicinal chemistry point of view. In this context, Goldberg et al. [14] from AstraZeneca shed light on “design guidelines and strategic goals when designing novel reagents for drug discovery projects”, and the 3D shape appears as one of the strategic goals for the design of reagents for drug discovery programs. The 3D shapes of the non-aromatic pyrrolidine and cyclopentane rings with the aromatic pyrrole ring are compared in Fig. 1. The different bond angles and lengths clearly suggest the flatness of the 2D structure of pyrrole and the 3D coverage of pyrrolidine and cyclopentane.

**Fig. 2:**
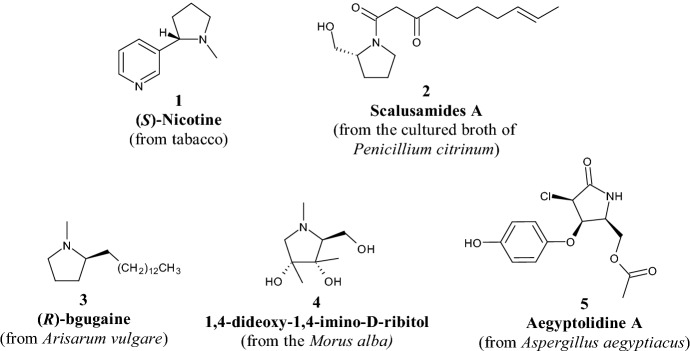
Representative structures of natural alkaloids **1–5**

A statistical analysis conducted by Tajabadi et al. [15] found that 70% of the 15,822 scaffolds belonging to natural products (NPs) are non-flat and represent an interesting resource for the design of new synthetic molecules [16]. Among such saturated ring systems, the pyrrolidine moiety is represented widely in NPs, especially in alkaloids isolated from plants or microorganisms [17–19] (Fig. 2) showing different biological activities, e.g. nicotine **1** (antioxidant, anti-inflammatory, antihyperglycemic properties), scalusamides A **2** and (*R*)-bgugaine **3** (antimicrobial, antifungal properties), 1,4-dideoxy-1,4-imino-d-ribitol **4** and aegyptolidine A **5** (anticancer properties). Therefore, it is no coincidence that the pyrrolidine nucleus is among the most preferred scaffolds in pharmaceutical science and drug design [20], as evidenced by the fact that it ranks first among the top five most common five-membered non-aromatic nitrogen heterocycles, appearing in 37 drugs approved by the United States (US) Food and Drug Administration (FDA) [21]. Moreover, pyrrolidine and its derivatives are used widely as ligands for transition metals, organocatalysts, and effective chiral controllers in asymmetric synthesis [22–24].

The great interest in the pyrrolidine scaffold has allowed chemists to explore new methods for its synthesis, thus bypassing conventional approaches. The application of microwave-assisted organic synthesis (MAOS) for the synthesis of pyrrolidines has had a strong impact, allowing synthetic efficiency to be increased, and also supporting the new era of green chemistry [25].

In this review, we highlight the interest of the chemistry community in pyrrolidine and its derivatives, including pyrrolizines, pyrrolidine-2-one, pyrrolidine-2,5-diones, and prolinol, incorporated in bioactive molecules with target selectivity published from 2015 to date. We have organized the review on the basis of the synthetic approach used: (1) construction of the ring from different cyclic or acyclic precursors, and (2) functionalization of preformed pyrrolidine rings, e.g., proline derivatives.

## Influence of Steric Factors on Biological Activity

**Fig. 3:**
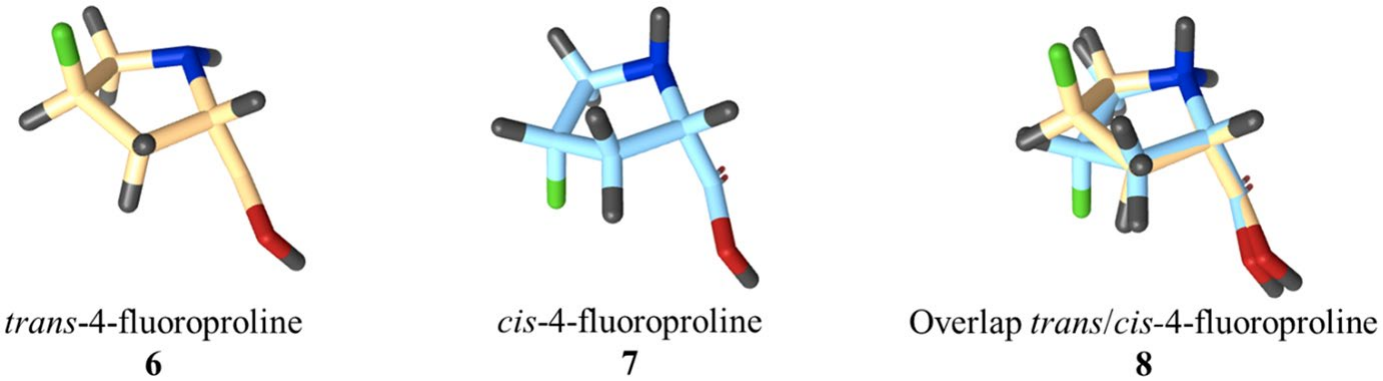
Exo and endo conformers of trans- and cis-4-fluoroproline **6** and **7**, respectively; trans/cis-4-fluoroproline **8** resulting from the superimposition of structures **6** and **7**

Contrary to the parent aromatic compound pyrrole, which is a common scaffold of several bioactive compounds [26–28], the great interest in synthetic pyrrolidines is also due to the presence of up to four stereogenic carbon atoms leading to up to 16 different stereoisomers [29]. In this regard, the non-essential amino acid l-proline, with one chiral center, is frequently employed as a building block to produce chiral compounds, and as a catalyst for successful stereoselective synthesis [30, 31]. Since proteins are enantioselective, the introduction of chiral centers would represent a purposive strategy for the generation of selective ligands. Moreover, knowing the absolute and relative configuration of chiral centers can allow the toxicity or inactivity of one of the enantiomers to be avoided, a concept regulated extensively by US FDA guidelines on the “Development of new stereoisomeric drugs” [32]. In contrast to the pyrrole ring, thanks to “pseudorotation”, an intrinsic property of saturated five-membered rings, pyrrolidines gain energetically advantageous conformations offering interesting 3D coverings [33]—a useful tool for the exploration of pharmacophore space via diversity-oriented synthesis (DOS) [34, 35]. Overall, based on the electronegativity of C-4 substituents, the pyrrolidine ring of proline acquires two specific conformations called C-4 (or Cγ) -exo and -endo envelope conformers. This means that puckering of the ring can be controlled easily through inductive and stereoelectronic factors. For instance, in the case of l-proline, the endo conformer is preferred, whereas trans-4-fluoroproline **6** and cis-4-fluoroproline **7** favor the exo and endo envelope conformation, respectively (Fig. 3). The overlap of trans/cis-4-fluoroproline **8** clearly shows the different folding of the molecules induced by the *R* or *S* configuration of the carbon bearing the fluorine atom. Conversely, C-4 alkylation of proline with a methyl or tert-butyl group is used to lock the opposite conformation [36].

### The Enantiopure Compound Shows Full Agonism Towards G-Protein Coupled Receptor 40

**Fig. 4:**
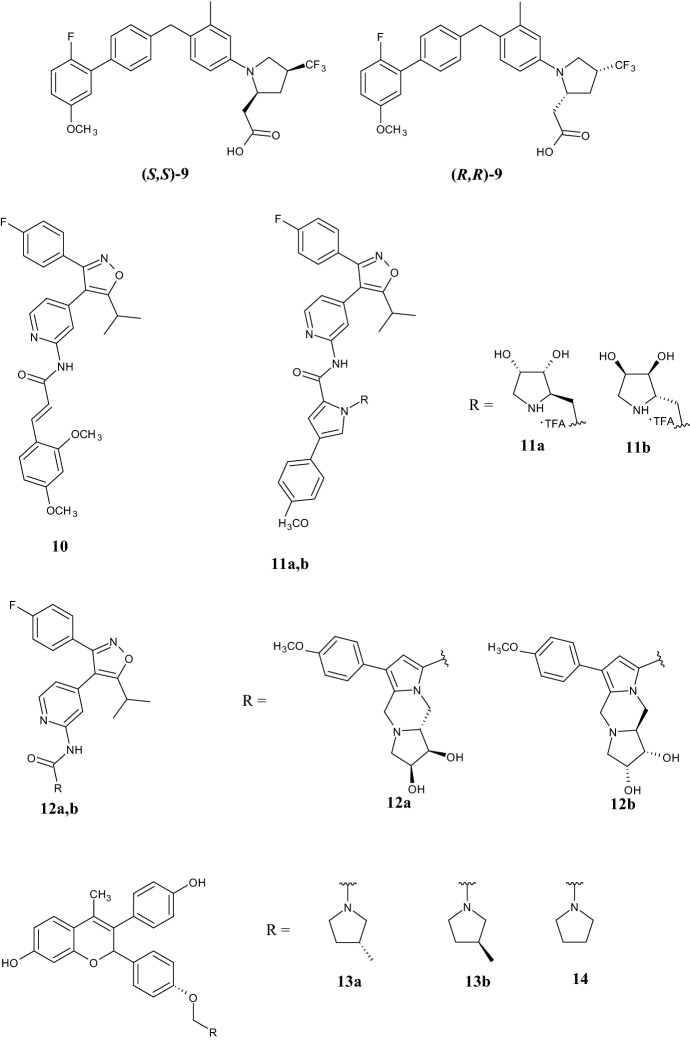
Stereospecific pyrrolidine derivatives (*R,R*)-**9**, (*S,S*)-**9**, **11a,b**, **12a,b**, **13a,b**, and **14**

Notably, Jurica et al. [37] synthesized a series of G-protein coupled receptor 40 (GRP40) agonists, like compounds (*R,R*)-**9** and (*S,S*)-**9**, for the treatment of type 2 diabetes (Fig. 4). They demonstrated that a cis-4*-*CF_3_ substituent on the pyrrolidine scaffold favors the pseudo-axial conformation of the groups in the other positions, as evaluated for the acetic acid group at position 2, which is the main pharmacophore for GRP40 agonists. By lead optimization, they observed that the enantiopure (*R*,*R*)-**9** derivative displayed full agonism, both in human GRP40 (hGRP40, 0.11 µM) and mouse GRP40 (mGRP40, 0.054 µM) due to the different binding mode compared to its enantiomer (*S*,*S*)-**9** (hGRP40 0.49 µM and mGRP40 2.4 µM), in addition to a better in vivo profile in lowering glucose plasma levels in mice tested in the oral glucose tolerance test at 0.3 or 1 mg kg^−1^ doses, and a dual mechanism of action including glucose-dependent insulin and GLP-1 secretion in vitro.

### The New Spatial Arrangement in the Crystallized Ligand–Protein Complex Promotes Selectivity for CK1γ

The role of steric factors was also investigated by Luxenburger et al. [38], who synthesized potent and selective CK1 kinase inhibitors exhibiting a chiral pyrrolidine scaffold, functionalizing the CK1γ inhibitor derivative **10** (Fig. 4). Interestingly, the new enantiopure hydroxyl-functionalized pyrrolidine derivatives **11a–b** (Fig. 4) showed selectivity for CK1 in the enzyme assay against a panel of 320 different kinases. To investigate the binding mode of the chiral moiety, X-ray crystallography was conducted. The crystal structure showed that, due to the incorporation of a methylene group through a spontaneously Pictet-Spengler cyclization, two new ligands (compounds **12a**,**b**) (Fig. 4) were formed. These compounds showed nanomolar activity against CK1γ and CK1ε (0.011 and 0.056 µM, and 0.024 and 0.196 µM, respectively), thus suggesting that further modifications should be made to investigate how the chiral moiety influences kinase inhibition.

### The Orientation of 3-*R*-Methylpyrrolidine is Responsible for a Pure Estrogen Receptor α Antagonist and Selective ER Degrader

In 2018, through biochemical and biophysical assays, coupled with high-resolution X-ray crystal structures, Fanning et al. [39] provided a molecular explanation of how the stereospecific orientation can change the binding mode of antiestrogen benzopyran derivatives **13a,b** and **14** (Fig. 4) within the hormone-binding pocket. Specifically, they showed that the orientation of 3-*R*-methylpyrrolidine **13a** was responsible for the compound being a pure ERα (estrogen receptor α) antagonist and a selective ER degrader (PA-SERD) for the treatment of breast cancer, opposite to the 3-*S*-methylpyrrolidine **13b** and the unsubstituted derivative **14**. This is due to the capability of the *R*-methyl group of compound **13a** to increase the mobility in the loop connecting helices 11 and 12 (H11–12 loop) of the ERα protein.

### Introduction of a *Cis*-3,4-Diphenylpyrrolidine Moiety Provides a Potent Inverse Agonist of RORγt

**Fig. 5:**
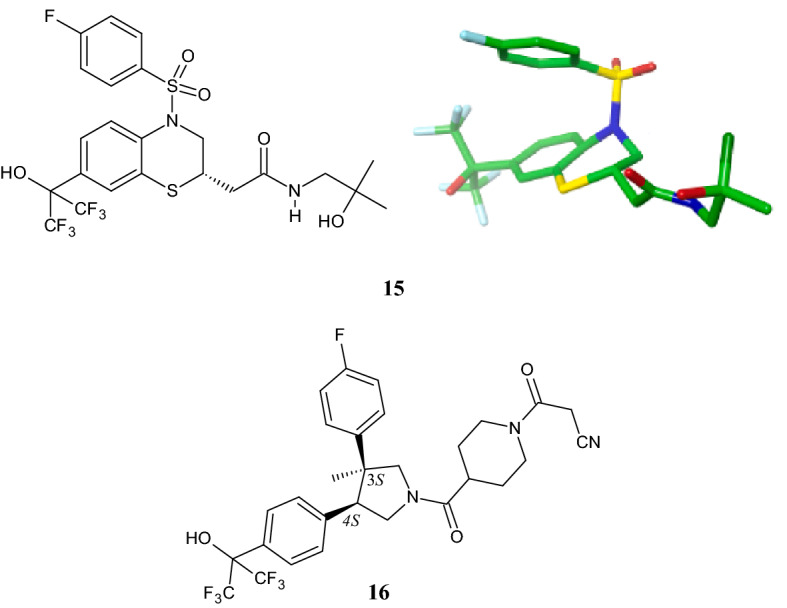
Structures of retinoic acid-related orphan receptor γ (RORγt) ligands **15** and its RORγt binding conformation (*top right*) [40] as well as **16**

Recently, Jiang et al. [40] demonstrated that replacing a non-stereochemical with a stereochemical group was beneficial for the activity of a new series of *cis*-3,4-diphenylpyrrolidine derivatives as inverse agonists of the retinoic acid-related orphan receptor γ (RORγt)—a splice variant of the nuclear hormone receptor subfamily RORγ involved in autoimmune diseases. The design of the new molecules started by studying the binding conformation of bicyclic sulfonamide **15** (Fig. 5), which showed excellent potency towards RORγt (12 nM) but suffered from undesirable activity against pregnane X receptor (PXR, EC_50_ = 144 nM, *Y*_max_ = 100%), which upregulates proteins involved in the detoxification and clearance of foreign toxic substances from the body. In the X-ray co-crystal structure of compound **15** in RORγt, the sulfonyl group assumes a pseudo-axial orientation with respect to the benzothiazine moiety, while the folding of the *para*-F-phenyl ring creates face-to-face *pi* stacking interactions with the benzene ring of the benzothiazine moiety, as shown in the upper panel of Fig. 5. This “U-shaped” conformation was maintained by replacing the sulphonyl group with the *cis*-3,4-diphenylpyrrolidine scaffold. Lead optimization led to piperidinyl carboxamide **16** (Fig. 5), which proved a balance between potency against RORγt with an EC_50_ of 61 nM and a considerably lower PXR activity (EC_50_ = 495, *Y*_max_ = 46%). Remarkably, (1) the cis-configuration accommodates the two phenyl rings close to the face-to-face stacked arrangement, (2) the hexafluoroisopropyl alcohol moiety establishes a hydrogen bonding interaction between the hydroxy group and His479 of RORγt, (3) transposition of the C3 methyl group in C4 position leads to the loss of RORγt potency. In addition, (4) pyrrolidines with an unsubstituted nitrogen were weak agonists compared with tertiary amines, and (5) a *para*-F substituent in the 3-phenyl ring improved EC_50_ values.

### Basicity and Nucleophilicity of the Pyrrolidine Nucleus

While substituents at C-4 of proline affect the puckering of the ring, substituents at C-2 of pyrrolidine shift its basicity. In fact, the nitrogen atom of pyrrolidine, as a secondary amine, confers basicity to the scaffold. In this context, An et al. [41] investigated the basicity of a set of 28 pyrrolidines used as organocatalysts, showing that particularly charged substituents have a strong effect on the basicity. Lastly, due to its nucleophilicity, the pyrrolidine nitrogen represents a privileged position for substitutions, with 92% of all US FDA approved pyrrolidine drugs being substituted at the *N* −1 position [21].

### Spatial Characteristics Influencing the Biological Activities of Pyrrolidine Derivatives

In summary, the advantages of using the pyrrolidine scaffold in drug design are due to the unrestricted conformation of the ring, which can be controlled and locked by the appropriate choice of substituents. Indeed, inductive and stereoelectronic factors influence the puckering of the pyrrolidine ring and, consequently, its pharmacological efficacy. The following list summarizes the spatial dispositions of the pyrrolidine scaffold that affect the biological activity towards specific targets cited in this chapter:

- cis-4-CF_3_ substituent on the pyrrolidine scaffold endorses the pseudo-axial conformation of the groups in the other positions, leading compounds able of showing full agonism in both the human and mice GRP40 receptor;
- compared with 3-*S*-methylpyrrolidine or an unsubstituted pyrrolidine, 3-*R*-methylpyrrolidine promotes a pure ERα antagonist and selective ER degrader (PA-SERD) for the treatment of breast cancer;
- the introduction of a chiral pyrrolidine into molecules promotes selectivity towards CK1 receptors. However, the selectivity and potency for the CK1γ isoform is due to the methylene group at position 2 being involved in spontaneous intramolecular Pictet–Splenger cyclization during crystallization with the protein;
- a cis-3,4-diphenylpyrrolidine scaffold gives the molecule a “U-shape” conformation that is beneficial for inverse agonistic activity on the RORγt receptor.

## Pyrrolidine Derivatives Obtained by Ring Synthesis

### Pyrrolidines Obtained by 1,3-Dipolar Cycloadditions

**Fig. 6.**
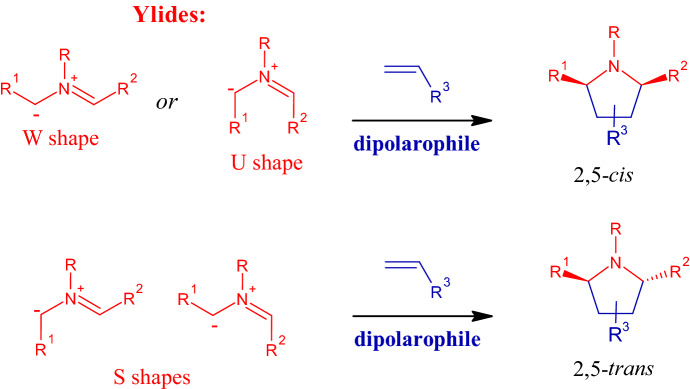
1,3-Dipolar cycloadditions to stereoselectively obtain pyrrolidine derivatives

A classical method for the preparation of five-membered heterocycles is the 1,3-dipolar cycloaddition [42] between a 1,3-dipole, such as a nitrone, an azide or an azomethine ylide, with a dipolarophile, typically an olefin, both of which are responsible for the regio- and stereoselectivity of the reaction [29, 43]. In particular, for synthetic pyrrolidines, the 1,3-dipolar cycloaddition reaction between nitrogen-based 1,3-dipole azomethine ylides with alkenyl dipolarophiles has been studied extensively. As shown in Fig. 6, stereoselectivity at positions 2 and 5 (R^1^ and R^2^) depends on the shape of the ylides, whereas stereochemistry in positions 3 and 4 correlates with the relative orientation of the substituents of the dipolarophile, leading to 3,4-cis- or 3,4-trans-substituted pyrrolidines [44].

**Fig. 7:**
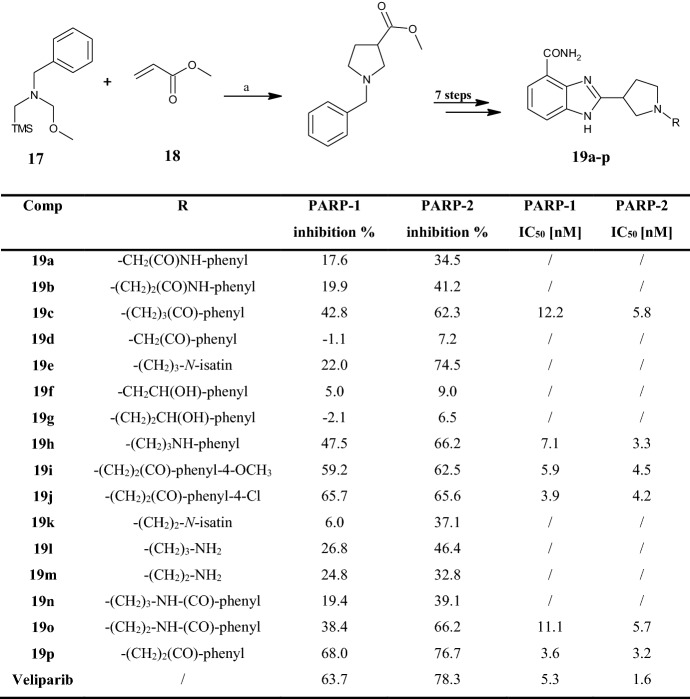
General synthetic scheme to benzimidazole carboxamides **19a–p**. R substituents and poly(adenosine 5′-diphosphate (ADP)-ribose) polymerase (PARPs) inhibition assay of compounds **19a–p**. PARP-1 and -2 inhibition (%) were evaluated at 10 nM. Reagents and conditions: *a* trifluoroacetic acid (TFA), dichloromethane (DCM), 17 h, room temperature (r.t.), yield: 96%

Exploiting the 1,3-dipolar cycloaddition process between *N*-(methoxymethyl)-*N*-(trimethylsilylmethyl)benzylamine **17** and methyl acrylate **18** (Fig. 7) under acid conditions in the presence of trifluoroacetic acid (TFA), Min et al. [45] synthesized a series of benzimidazole carboxamides **19a–p** bearing the pyrrolidine nucleus at position 2. In particular, they functionalized the pyrrolidine nitrogen by introducing different aromatic rings and tested the compounds as inhibitors of poly(ADP-ribose) polymerase-1 and -2 (PARP-1,-2), enzymes involved in the DNA damage repair process, as depicted in Fig. 7.

Out of 16 compounds, 6, namely **19c,h–j,o**,**p**, inhibited PARP-1 and -2 with IC_50_ values ≤10 nM. Structure–activity relationship (SAR) analysis revealed that the length of the alkyl chain and the presence of the carbonyl group greatly influence biological activity. Overall, the most promising derivatives were the phenyl ketone derivatives **19c**,**i**,**j**,**p**, rather than the *N*-phenylamine **19h** or the *N*-benzamide **19o** derivatives. Comparing the activity within the entire class of phenyl ketone derivatives bearing 2–4 carbon atoms in the alkyl chain (**19c,d,i,j**,**p**), compounds **19i,j**,**p** with the 3-carbon atom chain were the most active. Docking studies highlighted that the phenyl ketone fragments of compounds **19j** and **19p** would bind the three important amino acid residues Gly-863, Ser-904 and Glu-988 of PARP-1, instead of only Gly-863 and Ser-904 as in compounds **19c** and **19d**. Additionally, Van der Waals or hydrophobic interactions between the side chain of the compounds and the residues in the active site of PARP-1 were thought to facilitate the binding of these benzimidazole carboxamides. Electron donating or withdrawing groups in the para position of the benzene ring, as in compounds **19i** and **19j**, caused inhibitory activity similar to the reference compound veliparib, but with slightly higher IC_50_ values compared with the unsubstituted derivative **19p**. Reduction of the carbonyl group to a hydroxy group (**19f** and **19g**), the presence of hydrophilic amine chains (**19l** and **19m**), or the bulky phthalimide group (**19e** and **19k**), were detrimental for enzymatic inhibition. Cytotoxic assays against MDA-MB-436 (breast cancer) and CAPAN-1 (pancreatic cancer) cell lines confirmed **19j** as the most promising compound (IC_50_ 17.4 and 11.4 µM, respectively), followed by **19p** (IC_50_ 19.8 and 15.5 µM, respectively), which had IC_50_ values much lower than those of the reference compounds olaparib (IC_50_ 30.2 and 100 µM, respectively, in MDA-MB-436 and CAPAN-1 cells) and veliparib (IC_50_ 100 µM in both cell lines).

**Fig. 8:**
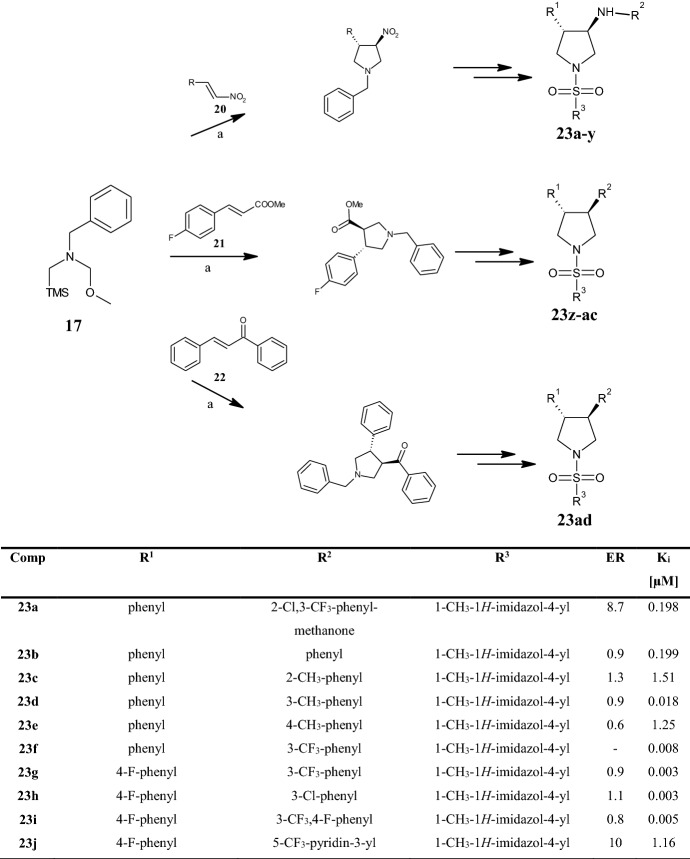

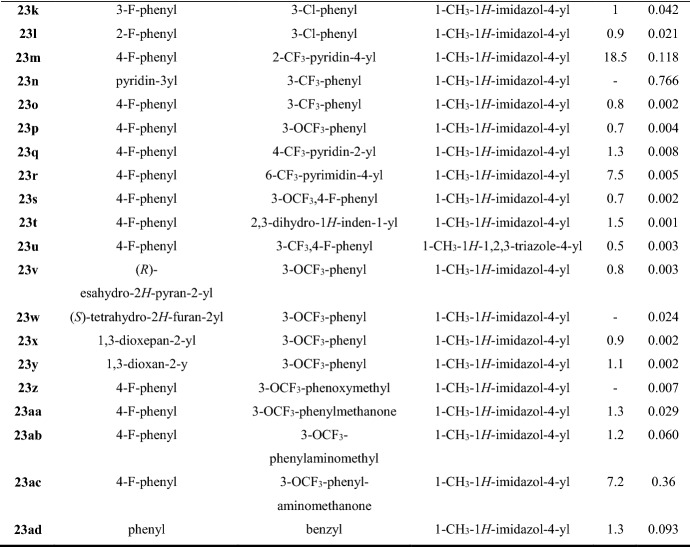
General synthetic scheme to pyrrolidine sulfonamides **23a–ad**. *ER* Efflux ratio values. *K*_i_ values towards hGLYT1 [46]. Reagents and conditions: *a* TFA (catalyst), DCM, r.t., overnight

The same dipolar reagent *N*-(methoxymethyl)-*N*-(trimethylsilylmethyl)benzylamine **17** was reacted with the nitrovinyl substrate **20**, methyl trans-4-fluorocinnamate **21**, and chalcone **22**, in the presence of a catalytic amount of TFA, by Wang et al. [46] to synthesize 3,4-disubstituted pyrrolidine sulfonamides **23a–y, 23z–ac**, and **23ad**, respectively (Fig. 8). All compounds were prepared as enantiomerically pure trans isomers and tested as selective glycine transporter-1 (GlyT1) competitive inhibitors. The major role of GlyT1 is to maintain glycine concentration below saturation level at the postsynaptic ionotropic glutaminergic *N*-methyl-d-aspartate (NMDA) receptor. Since the aetiology of schizophrenia has been linked to impaired glutamatergic neurotransmission involving the NMDA receptor, the development of GlyT1 inhibitors may represent a putative treatment for it and other disorders associated with NMDA receptor hypofunction. The new pyrrolidine sulfonamides **23a–ad** were synthesized based on the SAR investigation conducted on the reference compound **23a**. The latter displayed satisfactory GlyT1 inhibitory potency in vitro, with a* K*_i_ value of 0.198 µM, but a high efflux ratio (ER) of 8.7, indicating it as potential substrate of P-glycoprotein (P-gp)—the most relevant efflux transporter expressed in blood–brain barrier (BBB). The transformation of the sulfonamide moiety into amides, carbamides, tertiary amines, and urea groups, including the pyrrolidine nitrogen, produced inactive inhibitors of GlyT1 (structures not shown). The replacement of the benzoyl group of compound **23a** by aryl substituents (R^2^) gave excellent outcomes, providing new potent analogues such as **23d**,**f–i,k,l,o–t,y,z** with single or double digit nanomolar activity. Among these, the anilines **23d,g–i,k,l,o,p,s,v,x** also exhibited lower ER values and consequently less susceptibility to P-gp efflux. Comparable in vitro potency and ER values were obtained when replacing the 1-methylimidazole moiety by a 1-methyl-1,2,3-triazole substituent obtaining compound **23u**, which proved metabolically unstable. As reported in Fig. 8, the fluorophenyl substituents at position 3 (R^1^) of the pyrrolidine sulfonamides offered better in vitro potency and ER profile, followed by the unsubstituted phenyl ring (**23b–f**). Concerning R^2^ substituents, meta-substituted derivatives showed improved biological activity. Instead, the activity of compounds with heteroaromatic substituents in position 4 (**23j,m,q,r**) was influenced by the number and position of the nitrogen atoms of the heteroaromatic, as shown in Fig. 8. Finally, the analogue **23t** (R^2^ = indanyl) showed the best balance between potency and ER value (*K*_i_ = 0.001 μM, ER = 1.5).

**Fig. 9.**
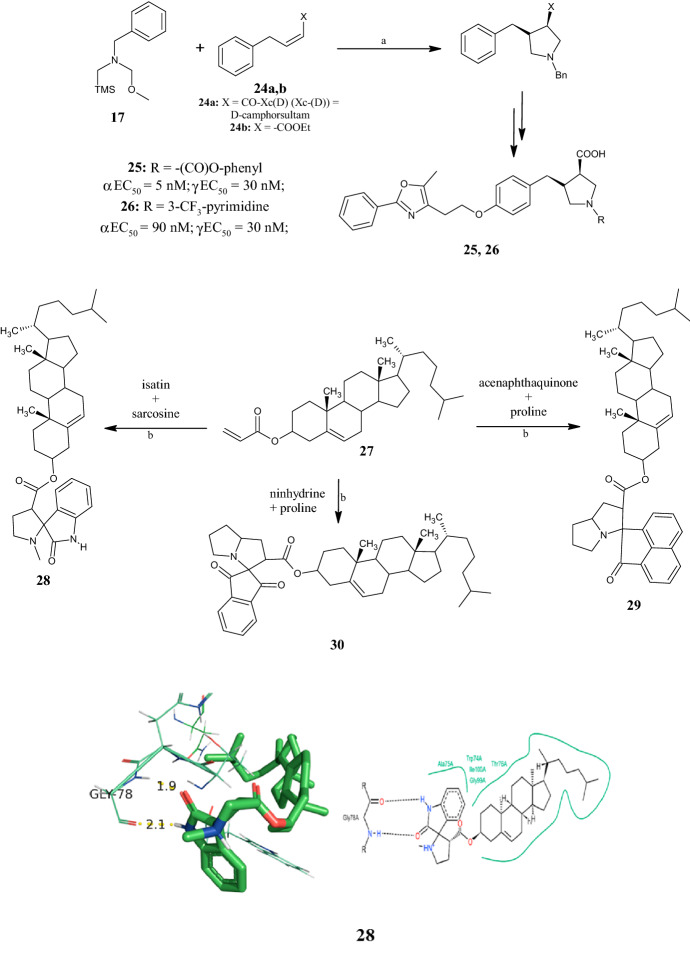
1,3-Dipolar cycloadditions to yield 4-benzylpyrrolidine-3-carboxylic acids **25** and **26**, synthesis of cholesterol-conjugated spiro-pyrrolidine/pyrrolizines **28–30**, and 3D/2D interaction diagrams of compound **28** with the active site of target receptor protein 1XFF [48]. Reagents and conditions: *a* TFA (1 M solution in DCM); **b** isopropyl alcohol (iPrOH), 2 h, under reflux, yields: 76% (**28**), 67% (**29**), 63% (**30**)

1,3-Dipolar cycloadditions between *N*-(methoxymethyl)-*N*-(trimethylsilylmethyl)benzylamine **17** and cis- or trans-alkenyl ester derivatives **24** (Fig. 9) in TFA, were used by Zhang et al. [47] to gain a series of 4-benzylpyrrolidine-3-carboxylic acid derivatives as potent agonists at peroxisome proliferator-activated receptors (PPARs). Among the new pyrrolidines, the cis-3R,4S-configured compounds **25** and **26** (Fig. 9) combine agonistic activity at PPARα and PPARγ, restoring glucose metabolism and ameliorating dyslipidaemia associated with type 2 diabetes. Compounds **25** and **26** displayed αEC_50_ and γEC_50_ values in the low nanomolar range (5–90 nM). In addition, compound **26** was efficient in lowering fasting glucose and triglyceride levels in diabetic db/db mice after oral administration of a 10 mg/kg dose once daily. SAR studies revealed that (1) the oxybenzyl pyrrolidine acid series offered the best balance of PPARα/γ functional activities, (2) the cis-configuration of the substituents in positions 3 and 4 of the pyrrolidine ring was preferred over the trans orientation, and (3) N-carbamoyl and N-aryl-substituted oxybenzyl pyrrolidine acid analogs provided potent balanced PPARα/γ dual agonists.

Conjugated systems with biological macromolecules, including carbohydrates, lipids, proteins, and nucleic acids, require that special attention is paid to the geometry of the molecules to be combined. Unlike the pyrrolidine scaffold, spiro-pyrrolidine molecules have rigid conformations that allow for easy incorporation into biological macromolecules, including cholesterol. For this purpose, Periyasami et al. [48] synthesized a small set of novel spiro-pyrrolidine/pyrrolizines by one-pot three-component reactions. Stereo- and regioselective reactions based on 1,3-dipolar cycloaddition between the dipolarophile C3-*β*-cholesterolacrylate **27** (Fig. 9) under reflux in *i*PrOH for 2 h with azomethidine ylides, in turn generated in situ by reaction isatin, acenaphthoquinone, or ninhydrine with the secondary amino acids sarcosine or proline, allowed the desired compounds to be obtained as single isomers. All compounds were evaluated for their in vitro antibacterial activity against a panel of four human pathogens, including *Vibrio cholerae*, *Proteus mirabilis*, *Micrococcus luteus*, and *Bacillus subtilis*. The most active were compounds **28**, **29**, and **30** (Fig. 9), which showed good antimicrobial activity, causing zones of growth inhibition in the performed disc diffusion assays ranging from 13.0 to 15.1 mm, at 50 µg/ml. With the aim of investigating their biological potential as glucosamine-6-phospate synthase (Glc-N-6P) inhibitors, the authors performed in silico molecular docking studies with compounds **28–30** in the enzyme’s active site (PDB ID: 1XFF). The results showed significant interactions with active site amino acids, revealing that the spiropyrrolidine moiety of compound **28** is engaged in two H-bond interactions involving its –NH and –CO groups with those of the aliphatic nonpolar amino acid Gly78 (Fig. 9), thus demonstrating its prominent role in the inhibition of Glc-N-6-P synthase and thus in the antibacterial activity of the compounds.

**Fig. 10.**
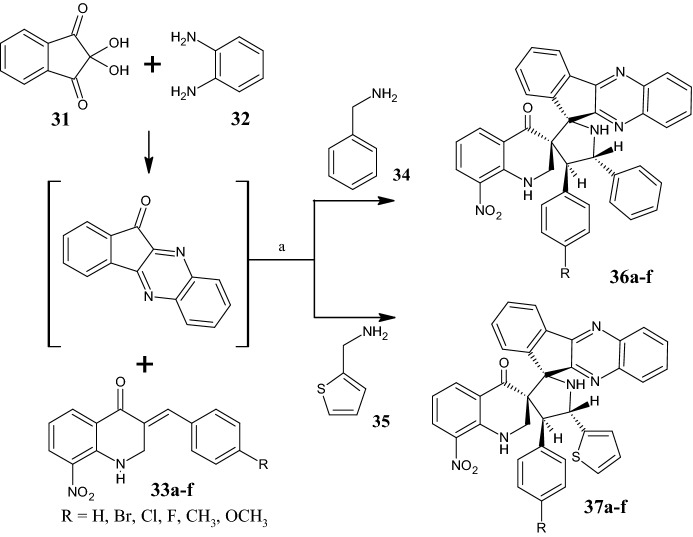
1,3-Dipolar cycloadditions to yield phenyl/thiophene dispiro indenoquinoxaline pyrrolidine quinolone derivatives **36a–f** and **37a–f**. Reagents and conditions: *a* MeOH, under reflux, 2–3 h, yields: 92–98% (**36a–f**) and 93–96% (**37a–f**)

By a one-pot multicomponent approach, Shyamsivappan et al. [49] synthesized phenyl/thiophene dispiro indenoquinoxaline pyrrolidine quinolone analogues, **36a–f** and **37a–f** (Fig. 10), via 1,3-dipolar cycloaddition reaction of (*E*)-3arylidine-8-nitro-2,3-dihydroquinolin-4(1*H*)-ones **33a–f**, ninhydrin **31**, *o*-phenylenediamine **32** and benzylamine **34**/thiophene methylamine **35** in MeOH under reflux for 2–3 h. All compounds were screened for anticancer activity against MCF-7 and HeLa cells, and only compound **37e** showed good biological activity, with IC_50_ values of 17 and 19 µM, respectively, comparable to those of the reference compound doxorubicin (16 and 18 µM against MCF-7 and HeLa cells, respectively). Overall, the thiophen-containing derivatives **37a–f** showed better activity against both cell lines (IC_50_ in the range of 17 and 28 µM against MCF-7, and 19 and 30 µM against HeLa) than their respective counterparts bearing the phenyl ring **36a–f** (IC_50_ in the range of 22 and 29 µM against MCF-7; and 26 and 37 µM against HeLa). SAR analysis indicated that compounds **36e,f,** and **37e,f**, characterized by electron donating groups, such as methoxy and methyl had lower IC_50_ values than other derivatives with electron-withdrawing groups (EWGs). Further investigation into the mechanism behind the anticancer activity revealed that compound **37e** induced apoptosis through intracellular reactive oxygen species (ROS)-mediated caspase-3 activation.

### Pyrrolidines Obtained by Pictet–Spengler-Oxidative Ring Contractions

**Fig. 11:**
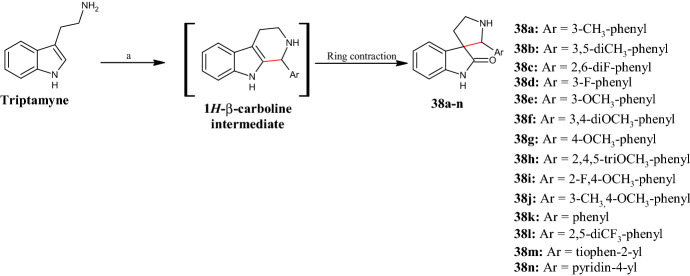
General synthetic scheme to spiro[pyrrolidine-3,3′-oxindoles] **38a–n**. Reagents and conditions: *a* ArCHO, NBS, water/tetrahydrofuran (THF), TFA (cat), 0 °C to r.t., yields: 45–94%

Moreover, spirocyclic motifs are emerging as an interesting feature for building block with low-molecular weight in the drug discovery field [50]. In 2016, Hati et al. [51] designed and synthesized a library of spiro[pyrrolidine-3,3′-oxindoles] **38a–n** (Fig. 11) as potential anti breast cancer agents with a dual activity against histone deacetylase 2 (HDAC2) and prohibitin 2 (PHB2) enzymes. HDAC2 is responsible for the deacetylation of lysine residues on the N-terminal part of core histones (H2A, H2B, H3 and H4) and provides a tag for epigenetic repression, thus playing a significant role in transcriptional regulation, cell cycle progression, and developmental events. PHB2 acts as a mediator of transcriptional repression by nuclear hormone receptors via recruitment of histone deacetylases and works like an ER-selective co-regulator, potentiating the inhibitory activities of anti-estrogens and repressing the activity of estrogens. Based on the DOS strategy, the new compounds **38a–n** were synthesized via a one-pot Pictet Spengler-oxidative ring contraction of tryptamine, in the presence of water as reactant, mediated by stoichiometric N-bromosuccinimide (NBS) as oxidant, and a catalytic amount of TFA, varying the aromatic functionality of the pyrrole domain. Among all compounds produced, **38d,h,i** inhibited the growth of human breast cancer cell line MCF-7 by inducing apoptotic cell death at low micromolar EC_50_ values (6.00, 4.01, and 3.53 µM, respectively). Chemical proteomics indicated HDAC2 and PHB2 as potential targets of the spiro[pyrrolidine-3,3-oxindoles] and molecular docking of the most active compound **38i** with HDAC2 (PDB ID: 4LY1) confirmed probable binding interactions. Overall, based on their cytotoxic effects, compounds characterized by electron-donating or weak EWGs on the phenyl ring showed higher MCF-7 cell growth inhibition rates compared with compounds bearing one or two strong EWGs.

### Pyrrolidines Obtained via Aminocyclizations

**Fig. 12:**
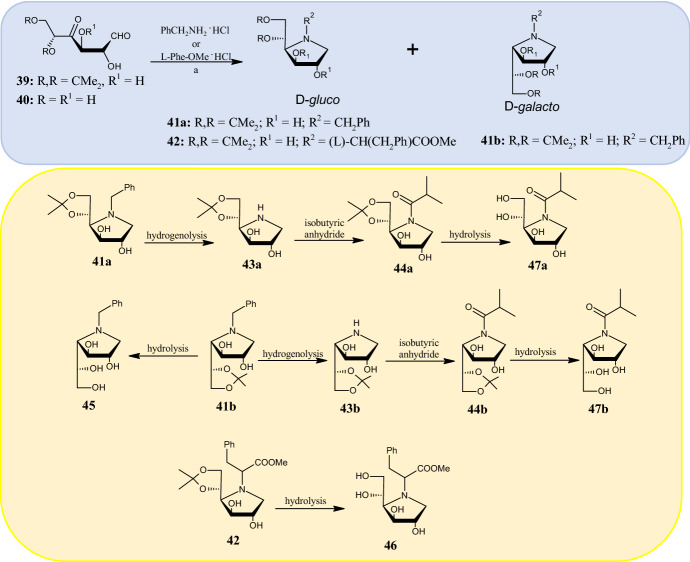
*Blue panel* Synthesis of diastereoisomers **41a,b** and **42** obtained by reaction of 5,6-*O*-isopropylidene-d-xylo-hexos-4-ulose **39** with benzylamine hydrochloride and l-phenylalanine methyl ester hydrochloride, respectively.* Yellow panel* Synthesis of final compounds **43a,b**, **44a,b, 45, 46** and **47a,b**. Reagents and conditions: *a* sodium cyanoborohydride (NaBH_3_CN), methanol (MeOH); 60 °C, 24–48 h, yields: 37% (**41a,b**), 44% (**42**)

Polyhydroxylated pyrrolidines, known as aza-sugars, are considered metabolically inert carbohydrates as they mimic the oxa-carbenium transition state from carbohydrate processing enzymes. Due to their central role in different biological activities, they are emerging as attractive compounds for the treatment of cancer and metabolic diseases. In 2019, using the double reductive amination reaction, Guazzelli et al. [52] developed a series of polyhydroxylated pyrrolidines, belonging to the diastereoisomeric d-glucose and d-galactose series, as dual-target inhibitors of the enzymes α-glucosidase (AG) and aldose reductase (ALR2). Although AG represents the most important enzyme for controlling plasma sugar concentration, pathological changes in nervous, renal, vascular, and ocular systems of diabetic patients are caused by activation of ALR2. Therefore, ideal antidiabetic agents should be able to simultaneously block the catalytic activity of AG and ALR2. Aminocyclization was conducted by reacting aldohexos-4-ulose derivatives **39** and **40** (Fig. 12) with amines, including benzylamine, benzhydrylamine, ammonia, l- or d-phenylalanine methyl ester, and sodium cyanoborohydride (NaBH_3_CN) as reducing agent, to obtain mixtures of d-glucose and d-galactose diastereoisomers. Among these, the authors isolated as pure compounds only diastereoisomers **41a,b** and **42** (Fig. 12) obtained by reaction of 5,6-O-isopropylidene-d-xylo-hexos-4-ulose **39** with benzylamine hydrochloride (for compounds **41a,b**) and l-phenylalanine methyl ester hydrochloride (for compound **42**). These three compounds were the starting material for the synthesis of other derivatives. Thus, through hydrogenolysis, compounds **43a,b** were obtained and were, in turn, reacted with isobutyric anhydride to give compounds **44a,b**. Finally, compounds **41b, 42**, and **44a,b** were subjected to hydrolysis to remove the 5,6-O-isopropylidene protecting group affording compounds **45**, **46**, **47a,b**, respectively, with enhanced hydrophilic character compared with the parent compounds. Among all, the d-galacto derivative **43b** was able to reduce cell death and restore the physiological levels of oxidative stress, showing a percentage of enzyme inhibition at 100 μM of 57.1% and 30.2% against ALR2 and AG, respectively.

**Fig. 13:**
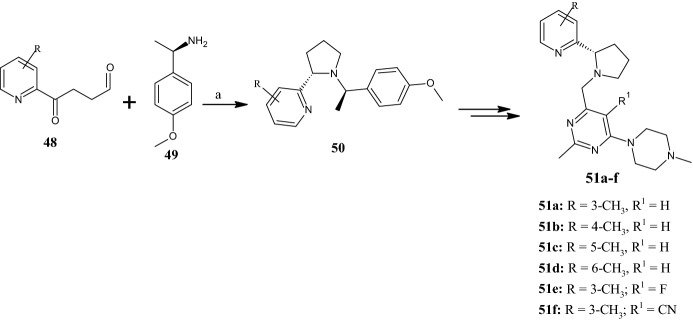
General synthetic scheme to pyrrolidines **51a–f**. Reagents and conditions: *a* sodium triacetoxyborohydride [NaBH(OAc)_3_], DCM, − 70 °C to r.t., overnight, yields: 21–45%

Recently, Li et al. [53] reported a series of (*S*)-pyrrolidines as CXCR4 chemokine receptor antagonists with antimetastatic activity. The synthesis of compounds **51a–f** (Fig. 13) was carried out in a multi-step manner in which intermediate pyrrolidine derivatives **50** were obtained by reaction of pyridin-2-yl-4-oxobutanal derivatives **48** with (*R*)-1-(4-methoxyphenyl)ethan-1-amine **49**. Among all compounds generated, **51a** with R^1^ = 3-CH_3_ showed excellent binding affinity to the CXCR4 receptor (IC_50_ = 79 nM competitively displacing fluorescent 12G5 antibody). Conversely, by shifting the methyl group to other positions in the pyridine ring, the IC_50_ value increased to 216, 278 and 2391 nM for compounds **51b**, **51c** and **51d**, respectively. Another feature of compound **51a** was its ability to inhibit CXCL12-induced cytosolic calcium flux (IC_50_ = 0.25 nM). In order to mitigate the overall basicity of the compounds, which can lead to issues such as hERG potassium channel inhibition, as well as CYP enzyme inhibition and phospholipidosis, the authors introduced a fluorine atom (compound **51e**) or a cyano group (compound **51f**) at the R^1^ position. However, this change greatly reduced the potency of compounds **51e** and **51f**, by 4- and 7-fold, respectively. Interestingly, the antimetastatic behavior of compound **51a** was also shown in an in vivo tumor metastasis test conducted in mice, which received compound **51a** intraperitoneally at a dose of 30 mg/kg.

### Pyrrolidine-2-Ones Obtained by Cyclizations

**Fig. 14:**
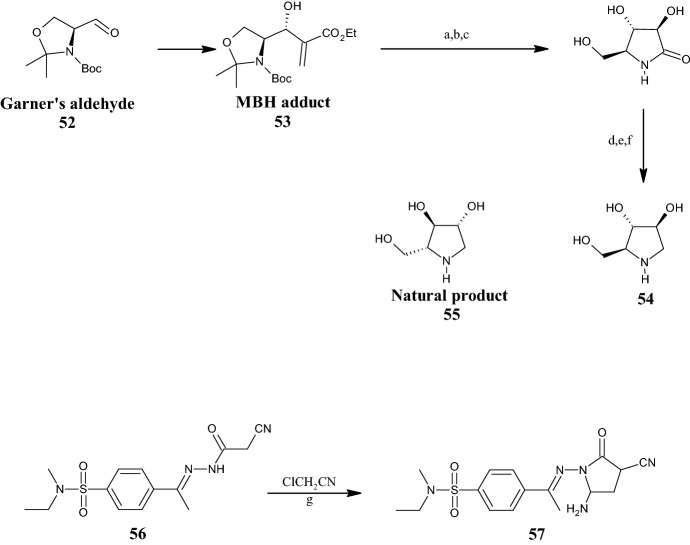
General synthetic schemes to polyhydroxylated pyrrolidines **54** and pyrrolidone **57**. Reagents and conditions: *a* (1) O_3_, MeOH, − 78 °C, 40 min; (2) DMS, 40 min, − 78 °C to r.t., not isolated; *b* [Zn(BH_4_)_2_], MeOH or DCM, − 20 °C, 2 h, yield: 74%; *c* TFA (2 equiv), DCM, 1 h, yield: 74%; *d* TBSCl, imidazole, DMF, 20 h, r.t., yield: 74%; *e* BH_3_. DMS, THF, 4 h, r.t., yield: 60%; *f* tetra-*n*-butylammonium fluoride (TBAF), THF, 12 h, r.t., yield: 99%. **g** triethylamine (TEA), 1,4-dioxane, 5 h, under reflux, yield: 77%

The interest in polyhydroxylated pyrrolidines led Da Silva et al. [54] to search for a short and alternative strategy to synthesize 1,4-dideoxy-1,4-imino-l-arabinitol **54** (Fig. 14). Compound **54** is a polyhydroxylated pyrrolidine that is a more potent α-glycosidase (AG) inhibitor than its natural enantiomer compound **55** (Fig. 14). For this reason, compound **54** is reputed to be a potential starting point for new antidiabetic and anticancer drugs. As shown in Fig. 14, the synthetic route proposed for the synthesis of **54** comprises six steps starting from a chiral Morita–Baylis–Hillman (MBH) adduct **53**, which in turn was prepared from Garner’s aldehyde **52**. Compound **54** was obtained from an intermediate pyrrolidone, which was synthesized by a linear three-step reaction sequence involving ozonolysis of the double bond of the MBH adduct **53**, followed by a stereoselective ketone reduction using zinc borohydride [Zn(BH_4_)_2_], concomitant *N*-double deprotection/O-deprotection with TFA, and a final amidation reaction (cyclization). Finally, the pyrrolidone intermediate was (1) silylated to decrease water solubility, (2) reduced with borane-dimethyl sulfide, and finally, (3) O-deprotected with tetra-*n*-butylammonium fluoride (TBAF) to afford compound **54**.

By intermolecular cyclization reactions between 2-cyanoacetamide derivative **56** and chloroacetonitrile, in the presence of triethylamine (TEA), under reflux in 1,4-dioxane for 5 h, Debbabi et al. [55] synthesized a series of *N*-ethyl-*N*-methyl benzenesulfonamides. Among these, derivative **57** (Fig. 14), characterized by a 5-amino-3-cyano-2-oxopyrrolidine core, showed antiproliferative activity against MCF-7 cells in the micromolar range (IC_50_ = 62.53 µM) and absence of cytotoxicity against normal fibroblasts of baby hamster kidney cell line (BHK). By docking studies, the activity of the compound was attributed to its binding to the dihydrofolate reductase (DHFR) (PDB ID: 4DFR).

### Pyrrolidine-2,5-Diones Obtained by Cyclizations

**Fig. 15:**
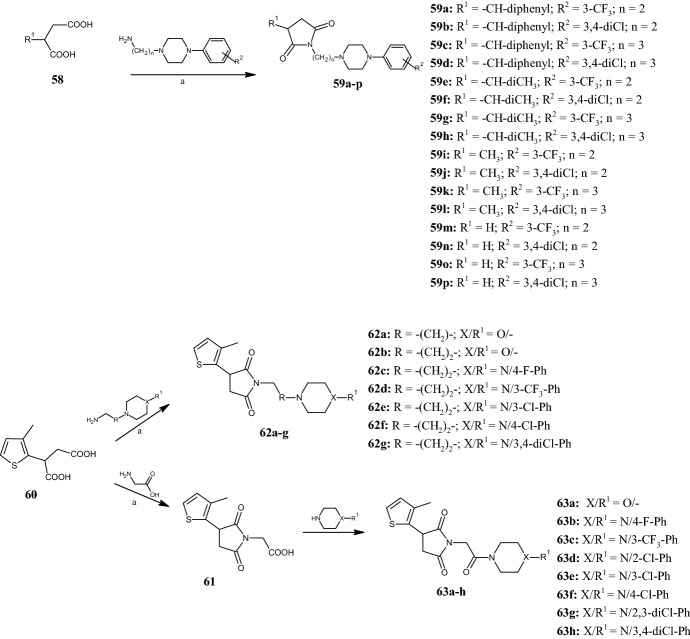
General synthetic routes to pyrrolidine-2,5-diones **59a–p**, **62a–g**, and **63a–h**. Reagents and conditions: *a* 180 °C, 1.5 h., yields: 60–82% (**59a–p**), 70% (**61**), 54–86 (**62a–g**)

Despite many years of research, it is still not clear how anticonvulsant drugs counteract seizures, but it is known that many of them interact with voltage-gated sodium channels (VGSCs) and voltage-gated calcium channels (VDCCs) in the central nervous system (CNS). In agreement with previous studies in which pyrrolidine-2,5-dione emerged as valuable scaffold in the treatment of epilepsy [56], in 2017, Rybka et al. [57] synthesized a library of 1,3-disubstituted pyrrolidine-2,5-diones **59a–p** (Fig. 15), obtained via cyclocondensations of dicarboxylic acids **58** with the properly substituted 1-(2-aminoethyl)- and 1-(3-aminopropyl)-4-arylpiperazines, at 180 °C for 1.5 h. Derivatives **59a–p** were administered intraperitoneally to mice and screened for their anticonvulsant activity by maximal electroshock (MES) and subcutaneous pentylenetetrazole (scPTZ) seizure tests in mice. Compounds **59j,n** showed good activity in both tests (MES ED_50_: 88.2 mg kg^−1^ and 101.5 mg kg^−1^, respectively; scPTZ ED_50_: 65.7 mg kg^−1^ and 59.7 mg kg^−1^, respectively), indicating that they are able to prevent various kinds of seizures by blocking the sodium channel with higher affinity than phenytoin. SAR analysis revealed that the anticonvulsant activity is affected strongly by substituents at position 3 of the pyrrolidine-2,5-dione scaffold. By scPTZ test, 3-benzhydryl **59a–d** and 3-isopropyl **59e–h** derivatives showed the most favorable protection in the scPTZ test, whilst 3-methyl **59i–l** and unsubstituted **59m–p** derivatives were more active in the MES test. Derivatives with a phenylpiperazine moiety bearing a 3-trifluoromethyl group were most active in the MES test, whilst 3,4-dichlorophenylpiperazines were active in both the MES and scPTZ tests. Finally, the increase in length of the alkyl chain resulted in derivatives that displayed quick onset and long-lasting anticonvulsant activity. A similar class of anticonvulsant and antinociceptive agents was also synthesized recently by Góra et al. [58], who designed hybrid derivatives of pyrrolidine-2,5-dione with the thiophene ring, compounds **62a–g** and **63a–h** (Fig. 15). While compounds **62a–g** were obtained by the reaction of 2-(3-methylthiophen-2-yl)succinic acid **60** with aminoalkylmorpholine or 1-(3-aminopropyl)-4-phenylpiperazine in one step, derivatives **63a–h** were obtained in two steps by the reaction of 2-(3-methylthiophen-2-yl)succinic acid **60** with aminoacetic acid to give intermediates **61**, which, in turn, underwent the coupling reaction. The best anticonvulsant activity was observed with compound **62b**, which displayed an ED_50_ value of 62.14 mg kg^−1^ in the MES test, compared with references ethosuximide (ED_50_ > 500 mg kg^−1^) and valproic acid (VPA) (ED_50_ 252.7 mg kg^−1^), and 75.59 mg kg^−1^ in the psychomotor seizure (6 Hz) test, compared with references ethosuximide (ED_50_ 221.7 mg kg^−1^) and VPA (ED_50_ 130.6 mg kg^−1^). In addition, four of the tested compounds (**62a**,**b**,**d**,**g**) revealed peripheral analgesic activity in the writhing test. In particular, compound **62g** was active at a dose of 30 mg/kg, similar to aspirin at the same dose. Compounds **62d** and **62g** also showed central analgesic activity in the hot-plate test at a dose of 30 mg kg^−1^. SAR analysis revealed that the acetamide moiety can extend anticonvulsant activity in both the MES and scPTZ tests.

**Fig. 16:**
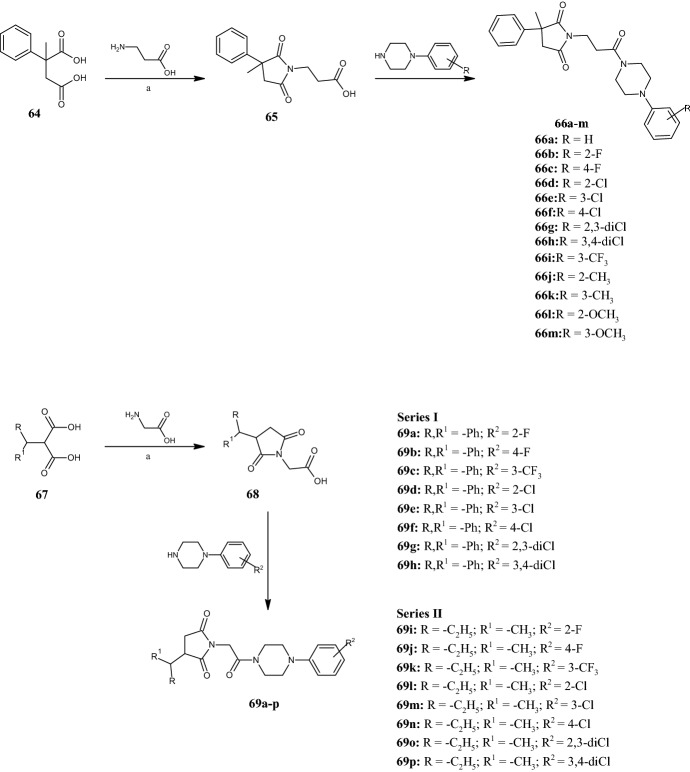
General synthetic routes to pyrrolidine-2,5-diones **66a–m** and **69a–p**. Reagents and conditions: *a* 180 °C, 1 h, yields: 68% (**65**), 70% (**68**, R, R^1^** = **phenyl), 36% (**68**, R = methyl, R^1^ = ethyl)

Later, Obniska et al. [59] expanded the same class of pyrrolidine-2,5-diones by substituting the thiophene ring with an unsubstituted phenyl moiety. The new compounds **66a–m** (Fig. 16) were obtained by the condensation of 2-methyl-2-phenyl succinic acid **64** with 3-aminopropanoic acid to give the intermediate derivatives **65**, at 180 °C for 1 h. This structural change did not improve the pharmacological activity compared with the previously mentioned compound **62b**. However, the activity of the most promising derivatives **66b**,**c**,**h** was better than the reference VPA and ethosuximide, both in the MES test with ED_50_ values of 78.3, 83.51 and 97.67 mg kg^−1^, respectively, and in the scPTZ test, in which only compound **66b** was active (ED_50_ 114.15 mg kg^−1^). Finally, in 2021, Góra et al. [60] studied the anticonvulsant properties of two new series of pyrrolidine-2,5-dione-acetamides **69a–p** (Fig. 16), exhibiting a benzhydryl or *sec*-butyl group in position 3 of the pyrrolidine ring. The compounds were synthesized via intermediates **68**, which were obtained by the reaction of succinic acid derivatives **67** with aminoacetic acid at 180 °C for 1 h. Among the tested compounds, derivative **69k** showed the best ED_50_ values of 80.38 mg kg^−1^ in the MES and 108.80 mg kg^−1^ in the 6 Hz tests, emerging as more effective than VPA. Summarizing the SAR analysis of this class of compounds, the activity appeared to be influenced by the substituent at position 3 of the pyrrolidine-2,5-dione ring, as well as the type of phenylpiperazine attached to the acetamide fragment. In particular, the non-aromatic substituent (*sec*-butyl) in position 3 of the pyrrolidine-2,5-dione ring and the 3-trifluoromethylphenylpiperazine fragment positively affect the anticonvulsant activity as for compound **69k**. Slightly less active than compound **69k** in the 6 Hz test was its 2-chlorophenylpiperazine analogue **69l** with a 1.2 fold higher ED_50_ value. On the other hand, the introduction of the benzhydryl group in position 3 of the pyrrolidine-2,5-dione ring and a 4-chloro- or 2,3-dichlorophenylpiperazine fragment (compounds **69f** and **69g**) increased the activity in the scPTZ test (data not shown).

**Fig. 17:**
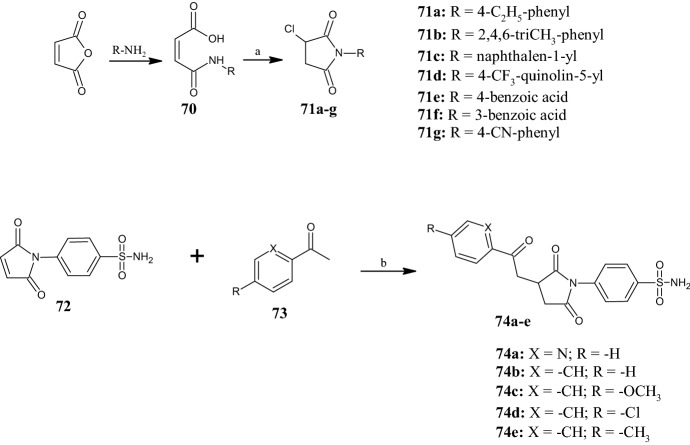
General synthesis of pyrrolidine-2,5-diones **71a–g** and **74a–e**. Reagents and conditions: *a* SOCl_2_, under reflux, 5–8 h, yields: 83–96%; *b* O*t*BU-l-threonine, 1,8-diazabicyclo[5.4.0]undec-7-ene (DBU), chloroform (CHCl_3_), r.t., yields: 51.3–63.2%

Pyrrolidine-2,5-dione is a versatile scaffold, as demonstrated by Oktay et al. [61] who prepared a series of 3-chloro-1-aryl pyrrolidine-2,5-diones evaluated for their inhibitory activity on the human physiologically relevant carbonic anhydrase (CA) isoenzymes hCA I and hCA II. Both isoenzymes are involved in several diseases, such as retinal and cerebral edema, glaucoma, and epilepsy. Therefore, their inhibition could be useful to counteract these issues. The synthetic route to obtain the 3-chloro-*N*-aryl pyrrolidine-2,5-dione derivatives **71a–g** (Fig. 17) started with the reaction between maleic anhydride and aromatic amines, with consequent opening of the ring to yield (*Z*)-4-oxo-4-(arylamino)but-2-enoic acid **70**, which, in turn afforded compounds **71a–g** by reaction with thionyl chloride (SOCl_2_) under reflux. All 3-chloro-1-aryl pyrrolidine-2,5-diones, except compound **71e**, were able to inhibit hCA I (*K*_i_ in the range of 23.27–36.83 nM) and hCA II (*K*_i_ in the range of 10.64 and 23.34 nM) with higher or comparable activity than the reference hCA inhibitor acetazolamide (AZA) (hCA I: *K*_i_ = 34.70 nM; hCA II: *K*_i_ = 31.93 nM). The most active compounds were derivatives **71d (**hCA I: *K*_i_ = 23.27 nM) and **71c** (hCA II: *K*_i_ = 10.64 nM), both decorated at the pyrrolidine-2,5-dione nitrogen atom with bicyclic scaffolds, such as quinoline and naphthalene.

As reported in the literature, cyclic imides, such as pyrrolidine-2,5-diones, possess interesting pharmacological properties due to the ability of the imide group to facilitate the crossing of biological membranes. Recently, Jan et al. [62] synthesized a series of pyrrolidine-2,5-dione derivatives **74a–e** (Fig. 17) as multitarget anti-inflammatory agents by applying a synthetic strategy based on Michael additions of ketones **73** to *N*-substituted maleimide **72**, at room temperature in the presence of O*t*BU-l-threonine and 1,8-diazabicyclo[5.4.0]undec-7-ene (DBU), using a self-assembled three-component system as organocatalyst. Biological activity was determined by in vitro assays, such as cyclooxygenase-1 (COX-1), cyclooxygenase-2 (COX-2), and 5-lipoxygenase (5-LOX), albumin denaturation and anti-protease assays, and in vivo in mice. In the in vitro assays, all compounds showed marked COX-2 inhibition compared with the reference diclofenac (IC_50_ = 10.05 µM) with IC_50_ values in the range of 0.98 and 8.94 µM. The most potent compound was **74e**, which showed selectivity for COX-2 over COX-1, with a selectivity index value (SI) [SI = IC_50_ (COX-1)/IC_50_ (COX-2)] of 31.5. In contrast, the SI values of compounds **74a–d** were 4.88, 11.5, 18.7 and 10.9, respectively. Furthermore, aryl ketone derivatives **74a** and **74e** showed excellent inhibition of human 5-LOX with IC_50_ values of 0.81 and 0.86 µM, respectively, which were slightly higher than the standard drug zileuton (IC_50_ = 0.63 µM). Finally, compound **74a** also induced albumin denaturation (IC_50_ = 5.36) and protease inhibition (IC_50_ = 13.39 µM). The in vivo test with the most promising compounds **74a** and **74e** revealed anti-inflammatory activity, which was ascertained with various mediators like histamine, bradykinin, prostaglandin and leukotriene. In addition, the same compounds showed safety in an acute toxicity study, in which the lethal dose (LD_50_) of both compounds in the experimental mice was approximately 1000 mg/kg. The SAR analysis revealed that the para-substituent on the phenyl ketone influences the biological activity. However, the replacement of the aryl ketone at position 3 of the pyrrolidine-2,5-dione nucleus with oxocycloalkyl/oxoalkyl groups (structure not shown) was detrimental to the anti-inflammatory properties.

### Pyrrolizines Obtained by Cyclizations

**Fig. 18:**
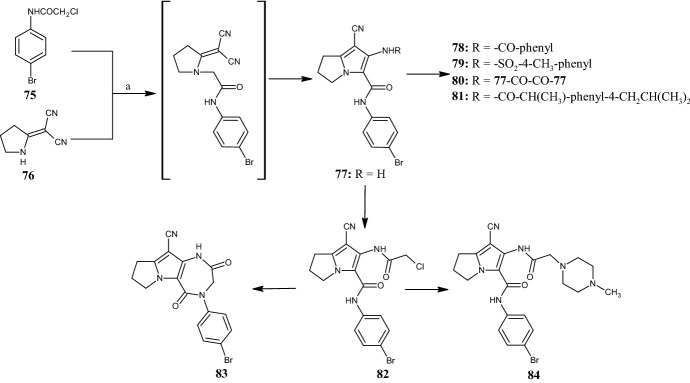
General synthetic scheme to pyrrolizine carboxamide derivatives **77–84**. Reagents and conditions: *a* K_2_CO_3_, acetone, under reflux, 24 h, yields: 68% (**77**)

In 2016, Gouda et al. [63] designed and synthetized a new series of pyrrolizine carboxamides **77–84** (Fig. 18) as dual cyclooxygenase (COX) and 5-LOX inhibitors with safer gastric profile. Pyrrolizine **77** was synthesized via the intramolecular cyclization of an intermediate obtained by reacting *N*-(4-bromophenyl)-2-chloroacetamide **75** with 2-(pyrrolidin-2-ylidene)malononitrile **76** under reflux in acetone for 24 h, and was used as starting material for the synthesis of derivatives **78–84**. All pyrrolizines were assayed for anti-inflammatory activity and showed IC_50_ values of 2.45–5.69 µM and 0.85–3.44 µM for COX-1 and COX-2, respectively. Furthermore, compound **77** exhibited higher anti-inflammatory and analgesic activities compared with ibuprofen. Upon NH_2_-acylation, its analogues, such as the 2-chloroacetyl (**82**) and the benzoyl derivative (**78**) were obtained. Introduction of the 4-tolylsulfonyl moiety into compound **79** further improved both biological activities. Conversely, the hybrid compound **81**, composed of the pyrrolizine precursor **77** and ibuprofen, did not show antiinflammatory activity and only a weak analgesic effect. 4-Methylpiperazine derivative **84**, the diazepine derivative **83**, and the dimer **80** showed lower anti-inflammatory and analgesic activities. Nevertheless, compounds **78** and **81** revealed better safety profiles than ibuprofen in acute ulcerogenicity and histopathological studies. Docking studies into COX-1 (PDB ID: 1EQG), COX-2 (PDB ID: 1CX2) and 5-LOX (PDB ID: 3O8Y, 3V99) showed that compound **79** fits well into the active sites of COX-1 and COX-2, whereas compound **79** exhibits the highest binding affinity for 5-LOX.

## Pyrrolidine Derivatives from Commercial Building Blocks

### Pyrrolidines from Proline

**Fig. 19:**
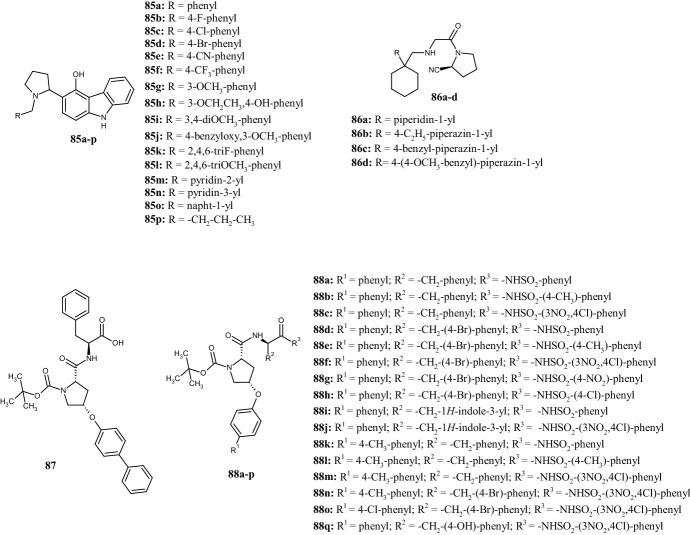
Molecular structures of pyrrolidinyl-carbazole derivatives **85a–p**, pyrrolidine-2-carbonitriles **86a–d**, Mcl-1 inhibitor **87**, and pyrrolidine-1-carboxylates **88a–p**

The interest in the pyrrolidine nucleus as skeleton of molecules with biological potential is endorsed by its similarity to the non-essential l-proline amino acid, which is used to obtain molecules with a specific stereochemistry. In 2017, Pannala et al. [64] developed a metal- and catalyst-free three-component decarboxylative coupling reaction of proline, aldehydes and 4-hydroxycarbazole to access pyrrolidinyl-carbazole derivatives **85a–p** (Fig. 19) with antiproliferative and antioxidant activities. The antiproliferative activity was evaluated by in vitro assays on three different cancer cell lines (MCF-7, MDA-MB-231, and lung cancer cells A549) and the results were compared with those obtained with the positive control doxorubicin. Overall, compounds **85b,c,k,p** showed the best cytotoxic activity against MCF-7 cells (IC_50_ 8.3–16.4 µM), whereas compound **85p** was also able to reduce the growth of A549 with an IC_50_ value of 15.7 µM. Furthermore, compounds **85b,f,h,j,m** showed higher radical scavenging abilities than ascorbic acid (IC_50_ 40.9 µM), with the best results displayed by compound **85 h** (IC_50_ = 27 µM) due to the presence of the hydroxy group on the phenyl ring. Docking analysis in the colchicine binding site (PDB ID: 1SA0) indicated that a methoxy-benzyloxy moiety (**85j**) and a trifluoromethyl group (**85f**) on the phenyl ring participate in hydrophobic binding interactions with several amino acid residues. Halogenated compounds **85b–d,k** displayed similar binding modes in the tubulin active site. Finally, a bulky naphthyl ring (**85o**) reduced the binding affinity.

The use of proline for the synthesis of pyrrolidine derivatives was also a strategic path for Nabil Aboul-Enein et al. [65], who synthesized a small class of antidiabetic compounds. The (*S*)-1-[(cyclohexylmethyl)glycyl]pyrrolidine-2-carbonitriles **86a–d** obtained (Fig. 19) were biologically evaluated for inhibitory activity toward dipeptidyl peptidase-4 (DPP4), a serine exopeptidase belonging to the S9B protein family, which cuts X-proline dipeptides from the N-terminus of polypeptides, such as chemokines, neuropeptides, and peptide hormones. As DPP4 is known to inactivate the incretin glucagon-like peptide-1 (GLP-1), the discovery of new DPP4 inhibitors is considered an indirect approach to increase GLP-1 levels and thus to manage diabetes mellitus type 2. The starting point for the synthesis of derivatives **86a–d** was (2*S*)-1-(chloroacetyl)pyrrolidine-2-carbonitrile, which could be obtained from *S*-proline via a chloroacetylation followed by an amidation of its carboxylate group and a final dehydration. The ability of compounds **86a–d** to inhibit the DPP4 enzyme was studied in diabetic mice that received the compounds orally at a dose of 100 mg/kg and the results were compared with those of the control group that received vildagliptin at the same dose. Serum DPP4 inhibition was evaluated 3 h after treatment. Among all, compounds **86b,c** inhibited DPP4 activity better (153% and 138%, respectively) than the control group (114%). In contrast, replacement of the piperazine ring with piperidine (**86a**) or (4-methoxybenzyl)piperazine (**86d**) reduced DPP4 inhibition to 85% and 105%, respectively. Docking studies confirmed a good binding affinity of compounds **86b,c** in the active site of DPP4 (PDB ID: 3W2T) in agreement with their anti-diabetic activity.

Starting from (2*S*,4*R*)-4-hydroxyproline, Wan et al. [66] designed and synthesized a new series of pyrrolidine derivatives based on compound **87**, which was described as potent inhibitor of myeloid cell leukemia-1 (Mcl-1) protein (*K*_i_ = 8.4 µM) (Fig. 19). Using a fluorescence polarization assay (FPA), these authors observed that the most potent compounds **88a–p** were characterized by phenyl groups at R^1^, aromatic or heteroaromatic rings at R^2^, and different benzenesulfonamides at R^3^. Compounds **88c** and **88f** showed the best binding affinities towards Mcl-1, with* K*_i_ values of 0.94 and 0.53 µM, respectively, slightly higher than the positive control gossypol (*K*_i_ = 0.39 µM) but very much lower than compound **87**. The replacement of phenyl or 4-bromophenyl at R^2^ with 3-indolyl and p-hydroxyphenyl (**88i,j,p**) was unfavorable for the biological activity. Finally, the replacement of the benzensulfonamide moiety with a hydroxy group and the introduction of linear chains or substituted phenyls instead of the 4-bromobenzyl group (structures not shown) resulted in the loss of binding affinity toward the Mcl-1 protein (*K*_i_ > 10 µM). Moreover, compound **88f** exhibited good antiproliferative activities against MDA-MB-231, PC-3 (prostate cell cancer), and K562 (chronic myeloid leukaemia) cell lines with IC_50_ values of 13.6, 10.7, and 23.0 µM, respectively.

**Fig. 20:**
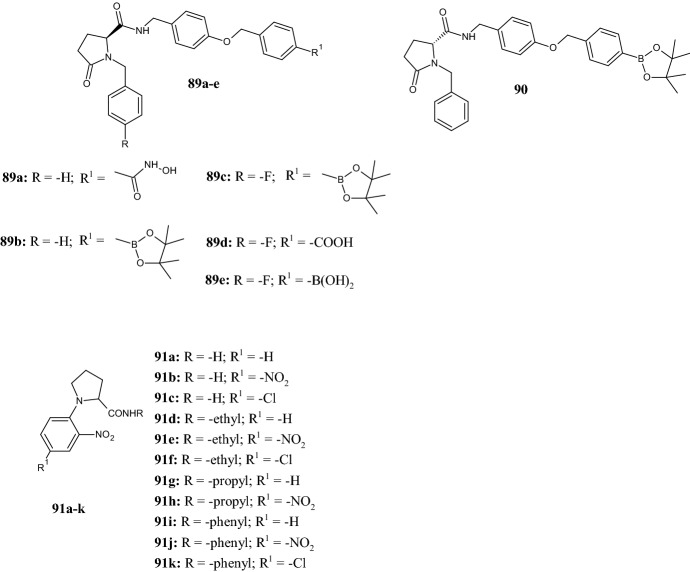
Molecular structures of pyroglutamic acid derivatives **89a–e** and **90** and *N*-(2′-nitrophenyl)pyrrolidine-2-carboxamides **91a–k**

In 2020, Gerokonstantis et al. [67] synthesized compounds **89a–e** and **90** (Fig. 20), which are potent inhibitors of autotaxin (ATX), a glycoprotein responsible for the hydrolysis of lysophosphatidylcholine (LPC) into bioactive lipid lysophosphatidic acid (LPA), whose upregulation is involved in pathological inflammatory conditions. The main scaffolds of the new derivatives were the natural amino acid *S*-proline, the naturally derived *S*-pyroglutamic acid ((2*S*)-5-oxopyrrolidine-2-carboxylic acid), and their enantiomers. In both cases, the new derivatives possess a benzyl type substituent at the pyrrolidine nitrogen, while the carboxylic group at position 5 is coupled to side chain via an amide bond containing a benzyl-ether type substituent, which bears carboxylate, methyl ester, sulfonamide, boronic ester, imidazole, hydroxamate, tetrazole, triazol, pyridine and boronic acid moieties in para-position (structures not shown). The in vitro assay showed that the pyroglutamic acid derivatives, including hydroxamic acid **89a** (IC_50_ 700 nM) and boronic acid derivatives **89b** (IC_50_ 50 nM), **89c** (IC_50_ 120 nM), **90** (IC_50_ 180 nM), and **89e** (IC_50_ 35 nM) were the most active compounds against the ATX enzyme, whereas only one out of six compounds—the carboxylic acid derivative **89d**—was the least active (IC_50_ 800 nM).

Antimicrobial peptides (AMPs) are small peptides with a wide range of inhibitory effects against bacteria and other pathogens. Pyrrhocoricin, apidaecin and drosocin are some examples of proline-rich antibacterial peptide family members able to bind the bacterial DnaK protein. In 2020, Odusami et al. [68] reported the synthesis of novel *N*-(2′-nitrophenyl)pyrrolidine-2-carboxamides **91a–k** (Fig. 20) with the aim of searching for amino acid analogues with antibacterial properties capable of mimicking antimicrobial peptides. The main structural features of compounds **91a–k** were the simultaneous presence of (1) a hydrophobic group provided by the phenyl ring, and (2) a cationic charge given by the amino group after its protonation, (3) different *N*′-substituents introduced to study the effect of conformational flexibility on the antimicrobial activity. The activity was evaluated both against Gram-positive [*Bacillus subtilis* (ATCC 19659), *Enterococcus faecalis* (ATCC 14506), *Mycobacterium smegmatis* (ATCC 14468), *Staphylococcus epidermidis* (ATCC 12228) and *Staphylococcus aureus* (ATCC 25923)] and Gram-negative [*Enterobacter cloacae* (ATCC 13047), *Escherichia coli* (ATCC 25922), *Proteus vulgaris* (ATCC 33420), *Klebsiella oxytoca* (ATCC 8724) and *Proteus mirabilis* (ATCC 7002)] bacterial strains, and the results were compared with those of reference compounds streptomycin and nalidixic acid. As assessed by the minimum inhibitory concentration (MIC) values, all the carboxamides **91a–k** were more potent against *S. aureus* and *E. cloacae* than the standard streptomycin (256 µg/ml and > 512 µg/ml, respectively), with compounds **91b** (15.6 µg/ml), **91c** and **91k** (62.5 µg/ml for both) being most potent against *S. aureus*, and compounds **91c** (62.5 µg/ml) and **91j** (31.3 µg/ml) being most potent against *E. cloacae*. Furthermore, the MIC values of compounds **91a–k** were lower than those found with nalidixic acid (≥ 500 µg/ml) against *E. faecalis*, *M. smegmatis*, *E. coli*, and *P. vulgaris* (MIC ≤ 250 µg/ml). Conversely, for other strains, the MICs of the carboxamides were higher than the ones of the reference compounds. SAR investigation showed that, in consideration of the *N*′-substituents, antibacterial activity increased in the order: *N*′-Et (**91d–f**) < *N*′-H (**91a–c**) < *N*′-Pr (**91g,h**) < *N*′-Ph (**91i–k**), whereas with the 4′-phenyl substituents, the activity increased in the order: 4′-PhH (**91a,d,g,i**) < 4′-PhCl (**91c** and **91f**) < 4′-PhNO_2_ (**91b**,**e**,**h**) except for the *N*′-phenyl carboxamides **91i–91k**, where **91k** (4′-Cl) > **91j** (4′-NO_2_) > **91i** (4′-H).

### Derivatives from Other Preformed Pyrrolidine Scaffolds

Given the interest of the scientific community in the pyrrolidine nucleus, many chemical industries have synthesized variously substituted pyrrolidines as building blocks for new drugs. Among these, pyrrolidine, pyrrolidin-2-one, pyrrolidine-2,5-dione, and prolinol scaffolds are very useful preformed rings for the synthesis of new bioactive compounds.

**Fig. 21:**
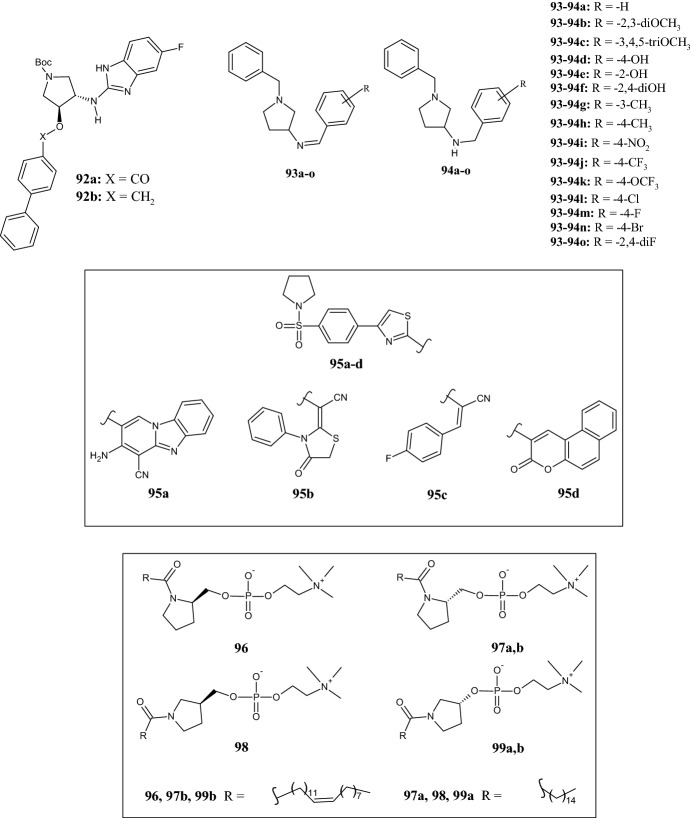
Molecular structures of pyrrolidine derivatives **92a,b**, Schiff bases **93a–o** and their reduced counterparts **94a-o**, benzenesulfonylpyrrolidines **95a–d**, and pyrrolidine-based 3-deoxysphingomyelins **96**, **97a,b**, **98**, **99a,b**

Beta-secretase 1 (BACE1) is the enzyme responsible for the proteolytic processing of the amyloid precursor protein (APP), which leads to the generation of amyloid-β (Aβ) peptides. The aggregation of Aβ in the brain of patients is responsible for the onset of Alzheimer’s disease (AD). Therefore, the development of treatments towards BACE1 could be a good strategy to fight this devastating neurodegenerative disease. In 2016, De Tran et al. [69] synthesized a library of (3*S*,4*S*)-4-aminopyrrolidine-3-ol derivatives **92a,b** (Fig. 21) as potential anti-AD agents with a target selectivity toward BACE1. An in vitro inhibition assay of BACE1 showed that compound **92a** was the most active, with an IC_50_ value of 0.05 µM. However, when replacing the carbonyl group of **92a** with a methylene unit as in the compound **92b**, the inhibition of BACE1 was approximately two times lower (IC_50_ = 0.12 µM). Unexpectedly, the opposite was observed in the cell-based assay ,where compound **92b** was more effective than compound **92a**, most probably due to a difference in cell permeability (IC_50_ values 1.7 versus 40 µM, respectively). Boc-deprotected derivatives having the pyrrolidine nitrogen atom unsubstituted or substituted with alkyl, acyl or sulfonyl groups were less active (structures not shown). A molecular docking study confirmed interactions with the BACE1 active site.

Novel compounds for the treatment of Alzheimer’s disease were also investigated by Choubey et al. [70], who synthesized multitargeted molecular hybrids of *N*-benzyl pyrrolidine derivatives, like imines **93a–o** and their reduced counterpart **94a–o** (Fig. 21). All compounds were studied for their inhibitory activity towards of cholinesterases (AChE and BChE) and BACE-1 in order to evaluate their multitarget profile. Overall, derivatives **94a–o** inhibited both cholinesterases as well as BACE-1 with greater potency than Schiff bases **93a–o**. SAR studies revealed that electron donating groups (EDGs) at the terminal phenyl ring elicit less potency with respect to acetylcholinesterase (AChE) inhibition than EWGs. Thus, derivatives bearing an EWG exhibited IC_50_ values of less than 1 µM, as observed for compounds **94k** (0.058 µM) and **94o** (0.069 µM), which were found to be almost as active as the reference donepezil (0.042 µM). A similar trend was observed for butyrylcholinesterase (BChE) inhibition. All the Schiff bases, with the exception of the compound **93j** containing a 4-CF_3_ group, showed a moderate inhibitory effect, whereas, among compounds **94a–o**, substitution with EWGs gave excellent BACE-1 inhibitors. Only in a few cases, such as for compounds **93j,k,o,** and **94c,d,f**,**i**, was the intended multitarget profile not observed.

Anticancer activity of pyrrolidine derivatives was studied by Bashandy et al. [71], who synthetized a new series of compounds characterized by the presence of a benzenesulfonylpyrrolidine moiety bearing a variously substituted 1,3-thiazole ring in position 4 of the phenyl ring. All compounds were tested for their in vitro antiproliferative activity against MCF-7 cells. Only a few compounds, namely **95a** (benzo[4,5]imidazo[1,2-*a*]pyridine), **95b** ((4-oxo-3-phenyl-1,3-thiazolidin-2-ylidene)ethanenitrile), **95c** ((4-fluorophenyl)-acrylonitrile), and **95d** (benzo[*f*]chromen-3-one) (Fig. 21), exhibited a slight improved activity compared to doxorubicin (IC_50_ = 68.6 μM), with IC_50_ values of 49.11, 48.01, 49.78 and 49.27 μM, respectively. In addition, molecular docking studies confirmed their ability to bind the DHFR active site (PDB ID: 4DFR).

In 2019, Hassan et al. [72] synthesized a series of pyrrolidine-based 3-deoxysphingomyelin analogues carrying various acyl chains (palmitoyl, palmitoleoyl, oleoyl, erucoyl, linoleoyl, and α-linolenoyl) at the pyrrolidine nitrogen atom and evaluated the compounds as antitumor agents against a panel of cancer cell lines including breast, non-small-cell lung, liver, and skin cancers. The most promising compounds were characterized by erucoyl (**96, 97b, 99b**) or palmitoyl (**97a, 98, 99a**) chains (Fig. 21). The best results were obtained for MCF-7 cells, which were more sensitive to the treatment with compounds **96**, **97b**, and **99b**, eliciting GI_50_ values at the micromolar level (15.7–24.8 µM). This effect was also confirmed in their study on the inhibition of Akt phosphorylation. In fact, compounds **97a**, **98**, and **99a** bearing identical acyl chains but exhibiting different stereochemistry, were equally active, with GI_50_ values of 21.1, 26.4, and 32.5 µM, respectively, confirming the stereochemistry as irrelevant for activity. Molecular docking studies established that the interaction with Akt (PDB ID: 3O96) may be the predominant mechanism of action of the 3-deoxysphingomyelin analogues tested.

**Fig. 22:**
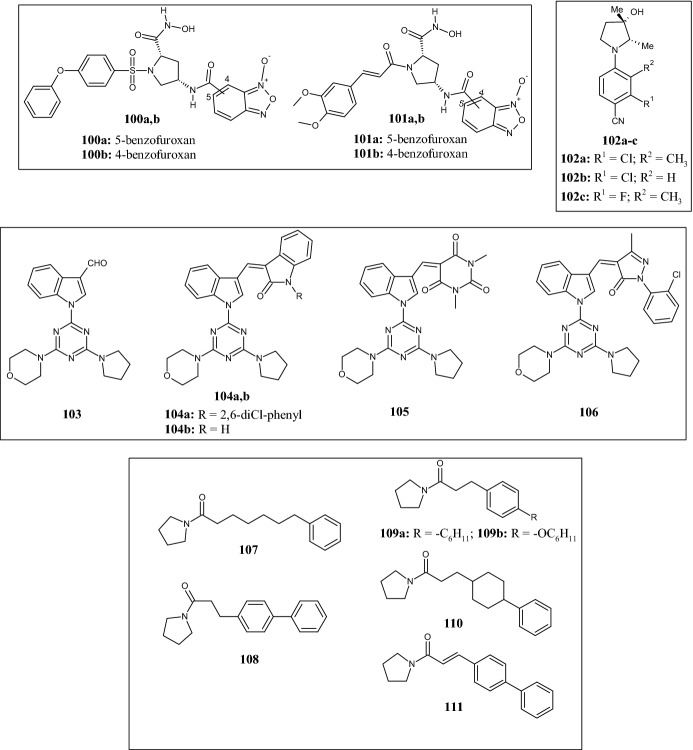
Molecular structures of hybrid benzofuroxan-based pyrrolidine hydroxamates **100a,b** and **101a,b**, pyrrolidine benzonitriles **102a–c**, hybrid pyrrolidine derivatives **103**, **104a,b**, **105, 106**, and pyrrolidine amides **107**, **108**, **109a,b**, **110**, **111**

In 2018, Zhang et al. [73] synthesized new hybrid benzofuroxan-based pyrrolidine hydroxamates carrying on the pyrrolidine nitrogen atom two different substituents: a 3-phenoxybenzenesulfonyl (**100a,b**) or a (3,4-dimethoxyphenyl)prop-2-enoly (**101a,b**) moiety (Fig. 22). All compounds elicited antiproliferative activity against several tumor cell lines, including A549, ES-2 (ovarian clear carcinoma cell), HeLa (cervix carcinoma), K562, MCF-7, and MDA-MB-231 (IC_50_ values of 3.56–25.64 µM), as well as NO-releasing capability (25.51–34.43 μM/l). The anticancer activity was ascribed to the inhibition of matrix metalloproteinases 2 and 9 (MMP-2 and MMP-9), as indicated by the reduced proteolytic activity after their isolation from treated cells. Among the phenoxybenzenesulfonamides, the 5-benzofuroxan **100a** showed higher MMP-2 and -9 inhibitory activity (IC_50_ values of 102 and 162 nM, respectively) than the 4-benzofuroxan analog **100b** (IC_50_ values of 182 and 242 nM, respectively), whereas the (3,4-dimethoxyphenyl)prop-2-enamide-5-benzofuroxan (**101a**) and the 4-benzofuroxan (**101b**) were less effective (IC_50_ values of 345–524 nM). Docking studies carried out in the active site of MMP-2 (PDB ID: 1HOV) with compound **101a** highlighted the ability of the hydroxamate group to chelate the catalytic zinc ion and both arylsulfonyl and benzofuroxan groups to create hydrogen bonds with amino acid residues.

Although polyhydroxylated pyrrolidines are important scaffolds of molecules for the treatment of metabolic diseases [52, 54], hydroxy groups could be subject to oxidation and conjugation reactions that would make the molecules metabolically unstable. In this regards, with the aim of modifying the pharmacokinetic profile, Asano et al. [74] synthesized 4-(pyrrolidin-1-yl)benzonitrile derivatives **102a–c** (Fig. 22) as selective androgen receptor modulators (SARMs) by optimizing the structure of previously reported 1-(4-cyano-1-naphthyl)-2,3-disubstituted pyrrolidine derivatives [75]. In particular, they introduced a methyl group at C-3 of the pyrrolidine ring that, due to its steric hindrance, should prevent metabolic instability, conferring better pharmacokinetic (PK) profiles than the parent 3-hydroxy compounds. Compounds **102b,c** had low cLogP values (< 3), showed better metabolic stability, and retained potent androgen receptor (AR) agonistic activity. In addition, 2-chlorobenzonitrile **102b** and 3-fluoro-2-methylbenzonitrile **102c** derivatives showed good bioavailability values (53.4 and 46.3%, respectively) and strong anabolic activity in levator ani muscle (> 300%), which was dose-dependent as demonstrated by in vivo studies on rats after oral administration. The X-ray co-crystal structure of **102c** bound to the AR LBD (PDB: 5T8J) highlighted a binding mode almost identical to that of the previously studied (2*S*,3*S*)-2-methyl-3-hydroxylpyrrolidine-2-chloro-3-methylbenzonitrile [75].

In 2018, Kaur et al. [76] designed and synthesized a library of hybrid molecules by combining triazine-indole with morpholine/piperidine/pyrrolidine and pyrazole/pyrimidine/oxindole moieties, as novel anti-inflammatory agents. All compounds were tested for inhibition of COX-1 and COX-2 and the results were compared with that of diclofenac, a COXs non-selective inhibitor, and celecoxib, a potent and selective COX-2 inhibitor. Of all compounds tested, pyrrolidine derivatives **103,104a,b,105,106** (Fig. 22) inhibited COX-2 with IC_50_ values in the range of 1–8 µM. The most potent was the compound **106**, characterized by a 1-(3-chlorophenyl)-3-methyl-2-pyrazolin-5-one moiety, with an IC_50_ value of 1 µM. The SI ratio (IC_50_ COX-1/IC_50_ COX-2) revealed compound **106** as selective for COX-2 (SI = 7), compared with compounds **106,104a,b,** and **105**, which were non-selective. In addition, docking studies highlighted a well-docked pose in the COX-2 active site showing H-bond and hydrophobic interactions. Surprisingly, compound **106** also decreased formalin-induced analgesia by 69%.

*N*-Acylethanolamine acid amidase (NAAA) is a lysosomal hydrolase that catalyzes the degradation of *N*-acylethanolamines into fatty acids and ethanolamine in animal tissues. Its inhibition is a therapeutic tool in several pathophysiological conditions, such as inflammation and immune disorders, as well as pain. In 2019, Zhou et al. [77] synthesized a new series of pyrrolidine amide derivatives as antiinflammatory agents and tested their selectivity towards NAAA and fatty acid amide hydrolase (FAAH), using rat NAAA (rNAAA) and rat FAAH (rFAAH) as animal model. The starting idea was to modify the linker chain and the terminal phenyl group of compounds **107** and **108** (Fig. 22), which were previously described as potent NAAA inhibitors (IC_50_ = 12.8 and 2.1 µM, respectively). The most active compounds were **109a**, (IC_50_ = 0.5 µM) **109b** (IC_50_ = 0.7 µM), **110** (IC_50_ = 0.48 µM), and **111** (IC_50_ = 1.5 µM) (Fig. 22) showing good NAAA inhibitory effects. Conversely, no relevant activity towards FAAH was found. Studies on the chemical space on compounds **107** and **108** indicated that the activity was lost upon substitution with CH_3_, Cl, and F in position 2 of the terminal phenyl ring of parent **107**. Instead, an improvement of activity was observed upon introduction of the same substituents at positions 3 or 4 (IC_50_ 3.7–9.6 µM), except for the 4-Cl derivative (IC_50_ 34.5 µM) (structures not shown). The same modifications on the terminal phenyl ring of compound **108** were detrimental for activity, most probably due to the conformationally restricted chain. Isosteric replacements of the distal phenyl ring with aromatic moieties such as 2-pyridyl, 3-pyridyl, 2-thienyl, and 3-thienyl, did not improve potency. This is in contrast to the introduction of a cyclohexyl ring as in compounds **109a** and **109b**, highlighting that (hetero)aromatic rings are less tolerated in the hydrophobic pocket of NAAA than aliphatic rings. Regarding the carbon chain between the pyrrolidine and the phenyl ring, the authors showed that flexible linkers led to a progressive increase in potency, except for compound **111**, which was well suited to the hydrophobic pocket of NAAA. Molecular docking studies (PDB ID:6DY2) suggested that **111** may inhibit NAAA via a reversible and competitive inhibition mechanism.

**Fig. 23:**
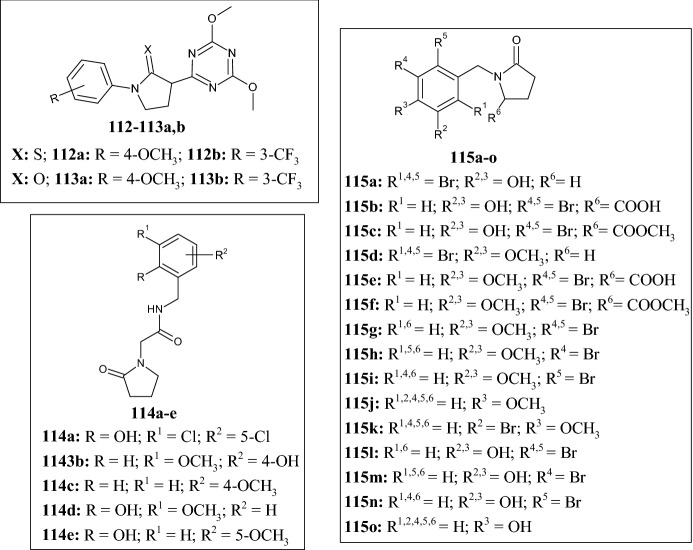
Molecular structures of triazine-pyrrolidine-2-thiones **112a,b** and pyrrolidin-2-ones **113a,b**, pyrrolidin-2-ones **114a–e**, and pyrrolidin-2-ones **115a–o**

As analogues of isoxazole compounds, in 2019 Lucescu et al. [78] synthesized and tested triazine-pyrrolidine-2-thiones **112a,b** (Fig. 23) on the human protein farnesyltransferase (FTase). This protein catalyses the addition of a C15-farnesyl lipid group to the cysteine residue located in the carboxy-terminal tetrapeptide motif of a variety of important substrate proteins playing an important role in malignant transformations including proliferation, apoptosis, angiogenesis, and metastasis. Compound **112a** showed good inhibitory activity toward the FTase enzyme with an IC_50_ of 3.82 µM, which was approximately 11-fold lower than that of compound **112b** (IC_50_ = 41.06 µM), highlighting the importance of the EWG on the phenyl ring. Interestingly, these two derivatives showed much better activity compared with the analogue in which pyrrolidine was replaced by the aromatic oxazole ring (structure not shown). In addition, the replacement of pyrrolidine-2-thione with pyrrolidin-2-one **113a,b** (Fig. 23) did not affect activity toward the FTase protein.

The pyrrolidine-2-one scaffold is a structural feature recurrent in antitumor agents, as demonstrated by Kumar et al. [79] who synthesized the novel derivatives **114a–e** (Fig. 23) by the reaction of substituted salicylaldehydes and 2-(2-oxopyrrolidin-1-yl)acetamide, leading to the respective Schiff base intermediates which were, in turn, reduced with sodium borohydride. Among all, compounds **114d** and **114e** showed the highest growth inhibition percentage against CCRF-CEM (acute lymphocytic leukemia) (**114d** = 73.15%) and NCI-H522 (non-small cell lung cancer) (**114e** = 41.8%) of the National Cancer Institute (NCI) panel. SAR analysis revealed that EWGs at positions 3 or 5 of the phenyl ring were unfavorable for antiproliferative activity compared with electron-donating methoxy groups. Hydroxy group introduction at position 2 (**114d** and **114e**) led to an increase in antiproliferative activity compared with compounds **114b** and **114c**. Docking analyses suggested binding to the podophyllotoxin pocket of the protein gamma tubulin (PDB ID: 1SA1) as a potential mechanism of action underlying the anticancer activity.

The versatility of the pyrrolidine-2-one scaffold was demonstrated by Rezai et al. [80], who synthesized novel *N*-benzyl-2-pyrrolidone derivatives **115a–o** (Fig. 23) as antioxidants and inhibitors of AChE and BChE. AChE is an ubiquitous enzyme of the serine hydrolases class responsible for hydrolyzing the neurotransmitter acetylcholine, together with the homologous BChE. AD is the most common form of dementia, characterized by loss of short-term memory, spatial disorientation, progressive loss of cognitive function, decreased intellect, and some other minor expressions. A common feature of the AD is the presence of AChE, which is usually related to the Aβ plaques and neurofibrillary tangles in the patient’s brain. Recent findings suggested that both Aβ and abnormally hyperphosphorylated tau protein (P-tau) may influence the AChE expression with the development of a vicious cycle of Aβ and P-tau dysregulation. In this context, AChE and BChE inhibitors can improve the cholinergic transmission, but with modest and temporary therapeutic effects. All compounds synthesized by the authors were able to inhibit the AChE enzyme at low nanomolar concentrations, with* K*_i_ values in the range of 2.60 and 16.36 nM. The most powerful AChE inhibitor was compound **115f** with a *K*_i_ value of 2.60 nM. Furthermore, all compounds inhibited BChE with *K*_i_ values in the range of 13.10 and 54.47 nM. Measuring the DPPH radical scavenging activity, compounds **115b,c,l–o** showed interesting antioxidant activity with half maximal radical scavenging concentrations (IC_50_ µg/ml) in the range of 4.71 and 53.30. The radical scavenger activity is affected by the phenolic fraction and varies according to the number and position of the hydroxy groups.

**Fig. 24:**
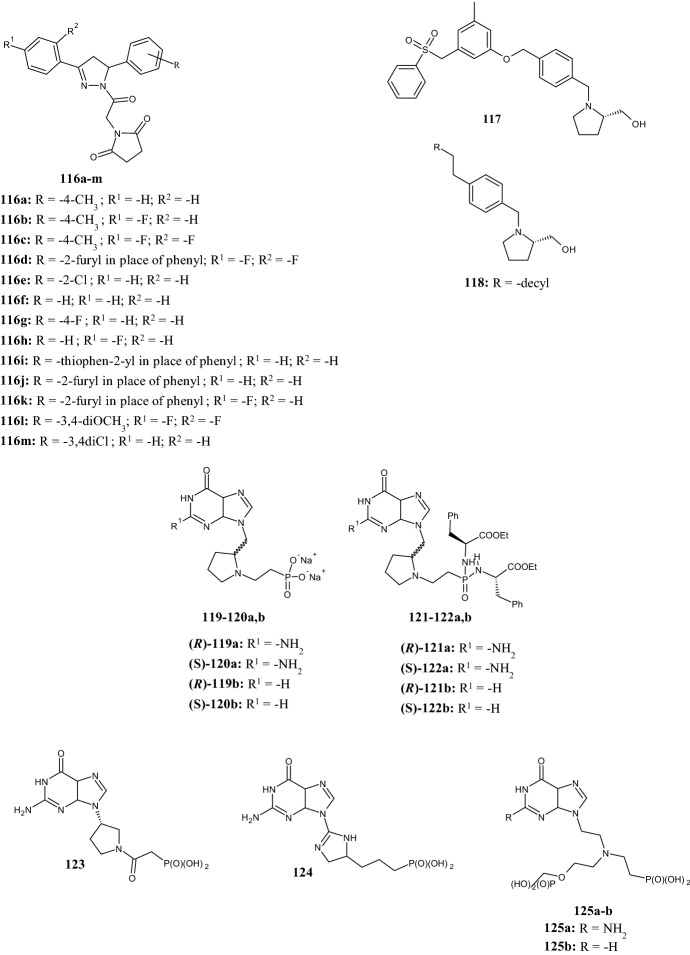
Molecular structures of hybrid pyrrolidine-2,5-diones **116a–m**, 2-(hydroxymethyl)pyrrolidines **117** and **118**, pyrrolidine phosphonates **119,120a,b**, and phosphoramidate prodrugs **121,122a,b**

To escape the problem of drug resistance, Tilekar et al. [81] recently published new pyrazoline-substituted pyrrolidine-2,5-dione hybrids **116a–m** (Fig. 24), with anticancer activity against MCF7, HT29, and K562 cancer cells. Compounds **116b**,**f**,**g**, showed nanomolar activity against MCF7 with IC_50_ values in the range of 0.42 and 0.78 µM. Compounds **116b** and **116m** showed also activity in the sub-micromolar range against HT29 cells (IC_50_ 0.92 µM and 0.39 µM, respectively). The cytotoxicity assay against K562 revealed that compounds **116a** and **116g** (IC_50_ 24.74 and 31.56 µM, respectively) were more potent than the reference compound pioglitazone (IC_50_ 40.3 µM). Further studies with compound **116b** demonstrated its ability to reduce the cell population in the G2/M phase and increase the cell population in the G0/G1 phase, as well as inhibition of the anti-apoptotic protein Bcl-2 in a dose-dependent manner.

To obtain potent dual sphingosine kinase 1/2 (SphK1/SphK2) inhibitors, Li et al. [82] synthesized a series of 2-(hydroxymethyl)pyrrolidines, based on SAR investigation of potent and selective inhibitors previously reported in the literature (compound **117**, SphK1-selective inhibitor) (Fig. 24). These enzymes are involved in the production of sphingosine 1-phosphate (S1P) from sphingosine and ATP, in a signalling pathway that is involved in cancer progression and immune cell chemiotaxis. While the 2-(hydroxymethyl)pyrrolidine scaffold is essential for hydrogen bonding with Asp178 in the binding pocket of SphK1, alteration of the aryl sulphonyl moiety changes the selectivity for SphK1 and SphK2. In this regard, the authors introduced various lipophilic substituents (i.e., alkyl, aryl, alkoxy, etc.) to the scaffold of compound **117** in place of the aryl sulphonyl moiety to mimic the sphingosine substrate of SphK1 and SphK2. The dodecyl analogue **118** proved to be the most potent dual SphK1/SphK2 inhibitor (SphK1 *K*_i_ = 0.679 μM, SphK2 *K*_i_ = 0.951 μM) compared with derivatives with a shorter alkyl tail. On the other hand, the diaryl ether, alkoxy, alkenyl, alkynyl analogues (structures not shown) showed no improvement in biological activity. Molecular docking studies highlighted that compound **118** fitted in the Sph binding pocket of SphK1 establishing hydrogen bonding of the 2-(hydroxymethyl)pyrrolidine moiety. Specifically, (1) the primary alcohol hydrogen bonded with Ser168 and (2) the tertiary nitrogen hydrogen bonded with Asp178. Similarly, hydrogen bonds between the pyrrolidine nitrogen and Asp308 as well as the hydroxyl group and Ser298 were observed in the homology model of SphK2 docked with **118**. The aliphatic dodecyl alkyl tail of compound **118** acquires a specific “J-shape”, generating hydrophobic interactions within the binding pocket of the enzyme.

Recently, Frydrych et al. [83] used commercially available d- and l-prolinol as the starting material for the synthesis of novel antimalarial agents, yielding new chiral compounds in which a pyrrolidine ring is incorporated in the linker connecting the purine base to the phosphonate group(s) (**119a,b** and **120a,b**) (Fig. 24). Compounds **119a,b** and **120a,b** were evaluated as inhibitors of the plasmodial hypoxanthine-guanine-(xanthine)-phosphoribosyltransferase [HG(X)PRT] of *Plasmodium falciparum*,* P. vivax* (HGPRT) and human HGPRT, and the results were compared with those of the previously published inhibitors **123, 124, 125a,b** (Fig. 24). The biological results did not show an improvement in activity over compounds **123, 124, 125a,b**. In fact, the new nucleotide analogues had *K*_i_ values in the range between 9 and > 50 mM for human HGPRT, 5–44 mM for *Pf*HGXPRT and between 20 and > 50 mM for *Pv*HGPRT, despite the increased flexibility achieved by the implementation of a CH_2_ group between the nucleobase and the pyrrolidine ring, which allows a free rotation. The same results were obtained when replacing the pyrrolidine nucleus with a six membered-ring, such as piperidine or piperazine (structures not showed). However, the phosphoramidate prodrugs **121a,b** and **122a,b** (Fig. 24), exhibited good antimalarial activity in a *P. falciparum*-infected human erythrocyte assay. In particular, the bisphosphoramidate prodrug **122a** was potent (IC_50_ = 2.5 µM) against the chloroquine resistant *P. falciparum* W2 strain, with low cytotoxicity in human hepatocellular liver carcinoma (HepG2) and normal human dermal fibroblasts (NHDF) cell lines at a concentration of 100 µM.

## Concluding Remarks

The present review is intended to provide significant support to medicinal chemists in the discovery of new biologically active pyrrolidine derivatives, providing a general overview of recent research concerning this scaffold, offering quick identification of the best synthetic route to apply. From this work, pyrrolidine emerges as a versatile scaffold found in molecules that exhibit a broad spectrum of biological activities. Pyrrolidines are useful to build compounds for fighting cancer and microbial infections, for metabolic diseases, as agents active in the CNS and for neurodegenerative diseases and immune disorders. Since three-dimensionality is an essential element of ligand–target interactions, the stereocenters present in pyrrolidine scaffolds would allow medicinal chemists to develop molecules with the most suitable configurations to fit into the ligand binding site of a target protein. In order to discover structurally novel compounds, the scaffold hopping strategy could be applied to the pyrrolidine core while maintaining its stereochemistry, together with exploration of the SAR of the most active pyrrolidine derivatives. We believe that the chemical versatility of the pyrrolidine nucleus and its ability to generate structural diversity could be essential to establish the clinical success of new bioactive molecules.

## Abbreviations

5-LOX: 5-Lipoxygenase
A549: Lung cancer cell line
AChE: Acetylcholinesterase
AD: Alzheimer’s disease
AG: α-Glucosidase
ALR2: Aldose reductase
APP: Amyloid precursor protein
AR: Androgen receptor
ATX: Autotaxin
AZA: Acetazolamide
Aβ: Amyloid-β
BACE1: Beta-secretase 1
BBB: Blood–brain barrier
BChE: Butyrylcholinesterase
BHK: Baby hamster kidney cell line
CAPAN-1: Pancreatic cancer cell line
Cas: Carbonic anhydrases
CC_50_: Concentration required to inhibit cell proliferation by 50%
CCRF-CEM: Acute lymphocytic leukemia
CNS: Central nervous system
COX-1: COX-2, cyclooxygenase-1, cyclooxygenase-2
DHFR: Dihydrofolate reductase
DNJ∙HCl: Deoxynojirimycin chlorohydrate
DOS: Diversity-oriented synthesis
DPP4: Dipeptidyl peptidase-4
α/γEC_50_: α/γ Half maximal effective concentration values
ED_50_: Median effective dose
ER: Efflux ratio
ER: Estrogen receptor
ES-2: Ovarian clear carcinoma cell
FAAH: Fatty acid amide hydrolase
FDA: Food and Drug Administration
FPA: Fluorescence polarization assay
FTase: Human protein farnesyltransferase
GI_50_: Growth inhibitory dose 50%
Glc-N-6P: Glucosamine-6-phospate synthase
GLP-1: Glucagon-like peptide-1
GlyT1: Glycine transporter-1
hCA I and II: Human isoenzymes I and II
HDAC2: Histone deacetylase 2
HeLa: Cervix carcinoma
hGLYT1: Human glycine transporter-1
IC_50_: Half maximal inhibitory concentration value
K562: Chronic myeloid leukaemia cell line
KIT: Tyrosine-protein kinase KIT
MBH: Morita–Baylis–Hillman
Mcl-1: Myeloid cell leukemia-1
MCF-7: Breast cancer cell line
MDA-MB-436: Breast carcinoma cell line
MES: Maximal electroshock
MMP-2: -9, Matrix metalloproteinases 2, -9
NAAA: *N*-Acylethanolamine acid amidase
NBS: *N*-Bromosuccinimide
NCI: National Cancer Institute
NCI-H522: Non-small cell lung cancer
NMDA: *N*-Methyl-d-aspartate
NP: Natural product
PARP-1-2: Poly(ADP-ribose) polymerase-1, -2
PXR: Pregnane X receptor
PC-3: Prostatic cancer cell line
PDB ID: Protein data bank identification
P-gp: P-glycoprotein
PHB2: Prohibitin 2
PK: Pharmacokinetics
PPARs: Peroxisome proliferator-activated receptors
P-tau: Tau protein
RORγt: Retinoic acid-related orphan receptor γ
SAR: Structure–activity relationship
SARMs: Selective androgen receptor modulators
scPTZ: Subcutaneous pentylenetetrazole
SI: Selectivity index
VDCCs: Voltage-gated calcium channels
VGSCs: Voltage-gated sodium channels
VPA: Valproic acid
